# Quick, Accurate, Smart: 3D Computer Vision Technology Helps Assessing Confined Animals’ Behaviour

**DOI:** 10.1371/journal.pone.0158748

**Published:** 2016-07-14

**Authors:** Shanis Barnard, Simone Calderara, Simone Pistocchi, Rita Cucchiara, Michele Podaliri-Vulpiani, Stefano Messori, Nicola Ferri

**Affiliations:** 1 Istituto Zooprofilattico Sperimentale dell’Abruzzo e del Molise, Teramo, Italy; 2 Engineering Department “Enzo Ferrari”, University of Modena and Reggio Emilia, Modena, Italy; University of Sydney, AUSTRALIA

## Abstract

Mankind directly controls the environment and lifestyles of several domestic species for purposes ranging from production and research to conservation and companionship. These environments and lifestyles may not offer these animals the best quality of life. Behaviour is a direct reflection of how the animal is coping with its environment. Behavioural indicators are thus among the preferred parameters to assess welfare. However, behavioural recording (usually from video) can be very time consuming and the accuracy and reliability of the output rely on the experience and background of the observers. The outburst of new video technology and computer image processing gives the basis for promising solutions. In this pilot study, we present a new prototype software able to automatically infer the behaviour of dogs housed in kennels from 3D visual data and through structured machine learning frameworks. Depth information acquired through 3D features, body part detection and training are the key elements that allow the machine to recognise postures, trajectories inside the kennel and patterns of movement that can be later labelled at convenience. The main innovation of the software is its ability to automatically cluster frequently observed temporal patterns of movement without any pre-set ethogram. Conversely, when common patterns are defined through training, a deviation from normal behaviour in time or between individuals could be assessed. The software accuracy in correctly detecting the dogs’ behaviour was checked through a validation process. An automatic behaviour recognition system, independent from human subjectivity, could add scientific knowledge on animals’ quality of life in confinement as well as saving time and resources. This 3D framework was designed to be invariant to the dog’s shape and size and could be extended to farm, laboratory and zoo quadrupeds in artificial housing. The computer vision technique applied to this software is innovative in non-human animal behaviour science. Further improvements and validation are needed, and future applications and limitations are discussed.

## Introduction

Monitoring the variation in animal behaviour is a commonly used technique, whether we are evaluating how a species is adapting to a changing captive environment or measuring the type and intensity of a reaction to an experimental challenge. As a direct reflection of the animal’s attempts to adapt to its surroundings, behaviour can be a good indicator of animal welfare [1]. Compared to biological parameters that require more handling (e.g. saliva/blood sampling for cortisol, heart rate devices), the non-invasiveness of behavioural measures can be considered an advantage. However, for practical application, reliable and exhaustive behaviour measurements suffer from time constraints and are sensitive to the observer experience, arbitrariness and accuracy. Scientific rigor in systematic observations demands that standardised protocols are applied with high repeatability and reproducibility. Modern advances in automatic video monitoring techniques and pattern recognition are opening new frontiers for on-line control and surveillance systems, citing Precision Livestock Farming as oneexample [2–4]. New software based on the analysis of two-dimensional video has been developed with the purpose of improving animal welfare. For example, automatic monitoring systems have been used to assess the behaviour of pregnant cows prior to calving [5], to record and analyse pig locomotor and aggressive behaviour in their barn environment [6,7], to assess the activity of broiler chickens with different gait scores or to quantify their behaviour inside furnished cages [8,9]. However, 2D top-view image analysis has intrinsic limitations such as the occlusion of some body-parts (e.g. legs) and the restriction in defining vertical movements (e.g. sitting, rearing). Recently, to overcome these limitations, researchers are moving toward 3D video monitoring using affordable equipment on the market such as the Kinect for Windows (Microsoft) for data acquisition [10–12]. Three-dimensional video monitoring and pattern recognition is a new frontier to the analysis of behaviour, overtaking traditional 2D tracking systems used to record mainly locomotor behaviour [7,10,13–17]. For example, Matsumoto et al. [11,12], recently used this approach to create a 3D model of the rat that allowed the study of social and mating interactions between rats and novel object recognition behaviours in a fully automatized way.

As the first domesticated species, the domestic dog (*Canis familiaris*) is well adapted to the human environment, which makes it an ideal subject in many research fields including animal welfare. Dog behaviour and locomotion are commonly recorded manually or semi-automatically using technological aids such as event recorders [18,19]. Although many ethological studies have been performed on this species, the use of automated behavioural analysis is still uncommon. Attempts have been made through the application of sensors as accelerometers and gyroscopes [20–22], GPS collars [23], or radiotelemeters [24]. Although new miniaturised technology offers small and light devices, these sensors are to a certain extent invasive for the animal wearing them. In contrast, video monitoring systems offer the advantage of being totally non-invasive.

Stray and free-roaming dogs are an increasing problem and concern in many developed and developing countries [25]. Confinement in shelters is among the most common technique for the control and management of these populations. Kennels and shelters are often overcrowded and an inappropriate environment or poor management can pose a serious risk to these animals’ welfare [18]. Research into a dogs’ ability to cope under confinement reveal kennels as a stressful environment, although individual variability plays a major role in adapting to it [18,26–30]. Behavioural indicators are widely used to assess dogs’ welfare in shelters [18,28,31,32]. However, as mentioned above, behavioural observation (through video recording or live scoring) may suffer from human error and can be very time consuming. This could be a limiting factor when resources are scarce [31,33,34]. Non-invasive video monitoring systems can potentially send in real time a great amount of data to a central logger with automatic analysis, limiting the amount of man-hours needed. Further, if running continuously, the system could allow the identification of rare behavioural patterns or behaviours that occur over many hours, which may be missed by observational sampling.

The aim of this pilot study was to test and validate a 3D image based software prototype, which to our knowledge is the first of its kind. This system is capable of automatically recognising the dog’s body parts and analysing its postures and behaviour in a confined environment. The software is able to track the movement and locomotor activity of the subject inside the pen. Furthermore, this software analyses and automatically describes recurrent spatio-temporal patterns of behaviours of the same or different animals using a multi-video cluster analysis. Limitations, suggested improvements, and future applications will be discussed.

## Materials and Methods

### Overview of the software

Three-dimensional videos were acquired both in the laboratory, with controlled artificial light and white background, and in a dog shelter. The behaviour of individual dogs was recorded using a Kinect depth camera for Windows (Microsoft). The collected frames were used to develop and compute a dog skeleton fitting seven body parts, i.e. head, torso, tail and four legs [35]. The depth images captured by the camera were used to define and remove all the planes of the kennel environment (e.g. fence, floor) isolating the blob of the dog (i.e. segmentation, see S1 Appendix, section 3). The depth image represents a cloud of points (pixels) that define the distance (with different scale of greys) from the camera to the surface of that object. In 3D space, that represents the surface of that object. A Structural Support Vector Machine (SSVM) was used to detect and classify each body part after exhaustive training. The generation of possible solutions for quadrupeds’ body structure was limited by anatomical constraints (e.g. the torso segment was forced to pass through the dog’s barycentre; the legs were constrained in the area below the torso). Also, the accuracy of the SSVM solution generations was checked by comparing it with a set of manual annotations of body parts on a subset of frames [35].

At present, the B.A.R.K. (Behavioural Automatic Recording in Kennels) software works live (real time scoring) or off-line using acquired openNI (.oni) files. The user is able to manually define the planes of the enclosed area, following few easy steps, allowing the software to isolate the dog from the background (i.e. segmentation see S1 Appendix, section 3). The body-parts of each individual are automatically extracted and generated from its blob image: the classifier chooses the best solutions from a number of outputs, before defining a skeleton that accurately fits that animal.

The B.A.R.K. software was designed to generalise the dog size and shape so it could be used with any subject. Potentially, this algorithm can be used with other quadruped species; the SSVM can be trained on new species to improve accuracy. The accuracy in detecting the dog skeleton can suffer from inadequate lighting (i.e. too much exposition) or occlusion due to very long-haired animals or very short legs (see discussion for limits of the software).

### Ethics statement

No special authorisation for use of animals (shelter dogs, *Canis familiaris*) in such behaviour/observational studies is required in Italy. The dog tested in the laboratory was owned by a PhD student, who volunteered to participate. The handler (i.e. owner) was present in the lab at all times, although not directly involved in the study. Since there was no threat of danger/harm, and no handling or direct contact with the dog (commands were given by the handler from a few meters away) no further ethical approval was needed. The land and shelter facility where the second part of the study was carried out were owned and managed by the affiliation institute (Istituto Zooprofilattico Sperimentale dell’Abruzzo e del Molise, Italy), which provided approval to conduct the study. Since animals were not subjected to invasive treatments nor did the protocols require an alteration of the dogs’ normal daily management routine, no other specific permissions were required for these locations/activities. All procedures were performed in full accordance with Italian legal regulations and the guidelines for the treatments of animals in behavioural research and teaching of the Association for the Study of Animal Behavior (ASAB). No endangered or protected species were included in this study.

### Animals and image acquisition

To implement the software with the features described in this paper, and to allow the validation procedure, a set of videos was acquired during two separate sessions.

In the first recording session a tester trained dog (Bearded collie mix, 7 y.o.) was introduced in a 3.5x3.5m laboratory room with controlled lighting condition. The handler asked the dog to respond to very simple command as ‘sit’, ‘stay’, ‘lie’ and ‘walk’. One Microsoft Kinect sensor was positioned on a tripod 1.5 m from the ground, 2 m in front of the dog.

In a second recording session, four individually housed dogs (large size mongrels, over 4 y.o.) were recorded in their residence pen, at a local rescue shelter sited in Teramo (Italy), for 30 min (between 11-13hours) without additional stimuli. The subjects varied in breed-type, coat length and colour and could be described as: one smooth-coated pointer-like dog with ticking white and brown hair, one wire-coated terrier-like dog with black and white patches, one herding/sheep-dog type with long white coat and one short-coated German Shepherd-like dog with bicolour black and red coat. The kennels had concrete flooring, were fenced with see-through wire mesh and completely covered with a roof to protect them from adverse weather condition. One Microsoft Kinect camera on a tripod, at 1.5 m from the ground, was positioned in front of the pen (3x3m) at a distance that allowed a view of the whole kennel perimeters.

### Structural classification of dog body parts and trajectories

Following our previous work in Pistocchi et al. [35] (S1 Supporting Article) we extracted the dog body-parts using a Structural Support Vector Machine (SSVM), a type of classifier widely used in computer science and pattern recognition. This field of study is called machine learning: classifiers are able to learn algorithms and create models from example inputs (i.e. training phase) and make prediction (expressed as outputs) on a pool of data without being explicitly programmed through static instructions (i.e. testing phase). SSVM represent an effective solution for structural learning problem and has been profitably applied in different computer vision context from segmentation [36] to tracking [37]. Hereinafter, few details will be given about the classifier algorithms and features, for more details please refer to the provided references.

During the training phase (supervised learning), the classifier learns to identify to which, of a set of categories, a new observation belongs (i.e. classification process). This is possible when a training set of data containing correctly categorised observations is available.

The classification is performed frame by frame where *X* is the input vector that represents the dog features and *Y* is a set of possible solutions (i.e. body parts labelling). The classifier learns the classification function h from the set of features *X* to the set of solutions *Y* on training samples of input-output pairs:

h:X→Y

At the time of testing only a sample *x* ∊ *X* is known from training, thus, the solution *y* ∊ *Y* (from the label space) is predicted through an inference problem.

To solve this problem, the SSVM uses a parameterisation of a Kernel function [38], which measures the similarity between two different input vectors and their corresponding solutions. In detail, during training the classifier maps every input-output pair *φ* (*x*_*i*_, *y*_*i*_) and generates a d-dimensional vector R^*d*^ (i.e. a real value vector in a d-dimensional space) based on the function

$$\varphi :XxY\to R^{d}.$$

Eventually, a scalar score function F (i.e. a function of real numbers) is learnt by the SSVM by finding the optimal set of parameters ***w*** that multiplies *φ* (*x*_*i*_, *y*_*i*_)

$$F(x,y)\to R=w^{T}\varphi \;(x_{},y)$$

This function measures the compatibility between (x, y) pairs, returning a high score value for well-matched pairs.

In addition to the classification of the dog’s body parts, we added the components of the motion vector of the dog barycentre (i.e. the centre of mass of the blob image), which is computed between two consecutive frames. The motion vector helps the classifier to orient the torso in the correct direction (i.e. front segment moving forward).

**Fig 1:**
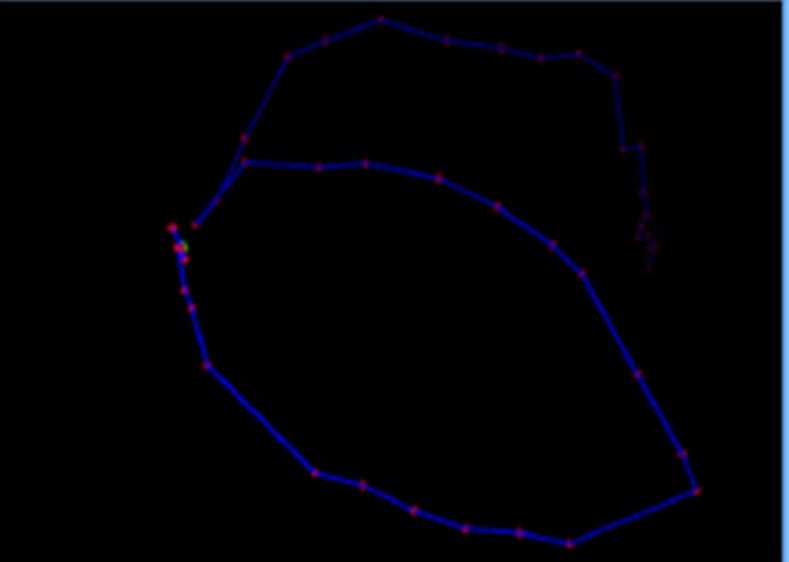
Example of a trajectory (top view) performed by a dog inside its pen. Fading lines are performed earlier, intense lines are more recent. Red dots represent the barycentre of the animal at a given instant. This bird eye view is achieved by re-projecting the acquired points through a homographic projection onto the pen’s ground plane. The projection matrix is computed automatically from the videos without manual calibration of the Kinect 3D sensor.

Spatio-temporal movements of the animal could be defined by recording frame by frame the position of the dog (i.e. coordinates of its barycentre) inside the pen. This allowed the inspection of the dog’s trajectories in space and time (Fig 1).

Thus, going back to our inference problem, to generate the dog skeleton, the classifier exploits a Kernel score function on a 16 dimensions real valued vector i.e. 7 solution segments (body-parts) and 2 values for every solution segment plus 2 components of the motion vector.

Computational burden are corrected for (e.g. by applying LaRank algorithm as explained in Pistocchi et. al [35]) to achieve an optimisation of the SSVM system.

At this stage, the system is able to work unsupervised without further training. The researcher would only need to manually select the 3D boundaries of the kennel. The blob of the dog (segmentation) is automatically extracted using motion information and the skeleton is generated (S1 Appendix section 3, 4).

It is important to mention that this system was developed to analyse the behaviour of the animal in terms of geometrical properties of the skeleton in space and time and not on the basis of a pre-defined ethogram. This means that the output has no ethological connotations attached to it; it is up to the researcher to interpret the data and assign behavioural categories and labels.

### Structural classification of dog postures

The structural classification of dogs’ postures helps the researcher to extract pre-defined behaviours of interest as an additional and optional feature.

The classifier can be trained on a new set of known data containing the behaviours of interest. For the purpose of this study, the prototype software was trained to label three main postures, but it could be trained to learn more if required. The same aforementioned structured learning method and algorithms, applied for the detection of the dog body-parts, were thus extended to dog postures. Hence, each structural classifier was trained independently on one of the selected postures (i.e. stand on four legs, sit and lie), each one defined by specific constraints on the generation of possible solutions (e.g. the classifier does not record the position of the legs when the dog is lying down). Through training, the classifier learns to match a given input vector representing the dog features (*X*) with a set of possible output solutions (*Y*) (i.e. the skeleton generated according to posture labelling) by the classification function

F:X,Y→R.

The final solutions are generated by maximising the compatibility between input-output pairs related to the postures as was done for the dog body-parts.

A total of 400 randomly selected frames were used for the dog posture training session. A schematic flow chart representing the segmentation and extraction method and the structural classifier solution generation can be found in S1 Appendix.

### Data mining and behavioural analysis

Given the information learned on postures and trajectories, the B.A.R.K. software can provide a number of outputs. All data can be exported in.*csv* format for further analysis.

Postures are automatically recorded in terms of duration of behaviours and frequency of occurrence. This information can be retrieved for the whole segment of video or for pre-defined time intervals (e.g. analysing the duration of behaviour within each 5 minute intervals).

**Fig 2:**
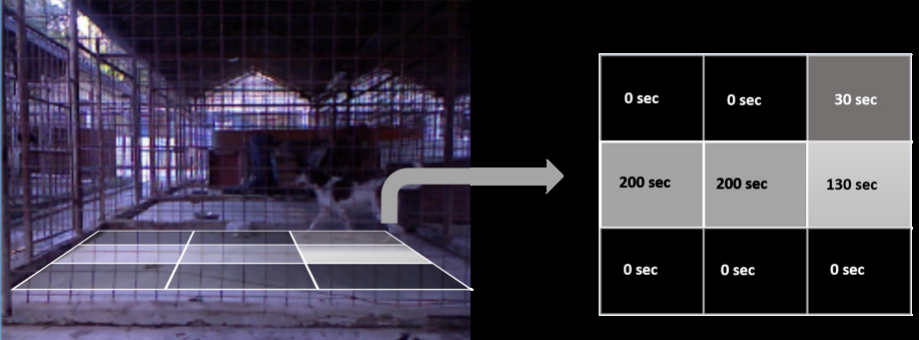
A grid divides the floor of the pen. The time (seconds) spent by the dog on each square is calculated. Different scales of grey also quantify the amount of time spent in each square (i.e. black = never entered the square, lighter shades = more time spent in that square).

The activity level of the animal during a given period of time, the time spent moving (seconds) and the distance travelled (estimated in meters) can be retrieved. Further, the floor of the pen is automatically divided into nine squares, and the time spent by the dog in each grid location can be calculated (Fig 2). A similar approach can be found in [16].

### Non-supervised clustering of behavioural patterns

Given a video (or set of videos) the B.A.R.K. is able to partition the clip in segments of pre-defined length (i.e. time intervals in seconds) and to perform an unsupervised analysis by clustering the segments according to similar patterns of behaviour that the animal is performing.

The system can analyse the changes in the behaviour of one animal in time (i.e. within the same video) or compare the behaviour of different animals from multiple videos or of the same animal in different time sequences.

**Fig 3:**
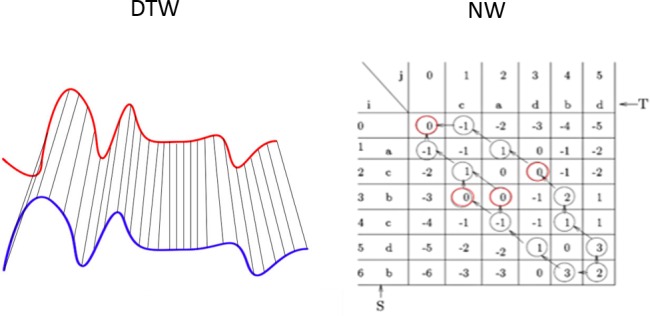
(a) Visual representation of the alignment of two sequences using the Dynamic Time Warping (DTW). The DTW stretches the sequences in time by matching the same point with several points of the compared time series. (b) The Needleman Wunsh (NW) algorithm substitutes the temporal stretch with gap elements (red circles in the table) inserting blank spaces instead of forcefully matching point. The alignment is achieved by arranging the two sequences in this table, the first sequence row-wise (T) and the second column-wise (S). The figure shows a score table for two hypothetical sub-sequences (i, j) and the alignment scores (numbers in cells) for each pair of elements forming the sequence (letters in head row and head column). Arrows show the warping path between the two series and consequently the final alignment. The optimal alignment score is in the bottom-right cell of the table.

The clustering is based on the distance between the fixed-length sequences computed on either trajectories (i.e. a sequence of dog coordinates) or postural representation (i.e. a temporal sequence of labelled body parts). However, there are cases where a pair of sequences may exhibit temporal shift and consequently a misalignment between corresponding elements. This limitation is overcome by the adoption of an elastic measure that aims at finding the best alignment between two sequences and eventually computing the distance (e.g. Dynamic Time Warping, Fig 3).

For aligning and comparing sequences we adopted the Needleman-Wunsch (NW) global alignment algorithm [39], also referred to as optimal matching algorithm. This algorithm was originally developed in bioinformatics to align any two sequences of proteins or nucleotides. Similarly, it applies to time series achieving the optimal alignment. Given two sequences T_i_ and T_j_, the global alignment is obtained by inserting spaces, either into or at the ends of the elements of each sequence, so that the length of the sequences are the same. By doing this, every symbol (or space) in one of the sequences is matched to a unique symbol (or space) in the other. Unfortunately, this algorithm can be very onerous in terms of computational complexity if the sequences are long. For this reason the NW algorithm uses a dynamic programming technique to reduce computational time to O (n_i_ n_j_), where n_i_ and n_j_ are the lengths of the two sub-sequences. Through dynamic programming, NW fills an alignment score table representing all the possible combinations of symbols and scores allowing to compute similarity scores efficiently (Fig 3).

In other words, the score table allows the efficient matching of all element pairs relying on the definition of a symbol-to-symbol distance (i.e. individual alignment scores based on Eq 1, Table 1). By assigning a scoring system to each individual pair, it is possible to define the best possible alignment between any two sequences. The maximum score among all possible alignments gives the optimal alignment score (i.e. described by Eq 2. Table 1).

**Table 1:** Global alignment and score table algorithms.

Eq	Description	Formula	Coding
**1**	Base condition for symbol-to-symbol matching in the score table	*V* (*a*; 0) = *Ω*(*Sa*;*i*;–); *V*(0; *b*) = *Ω* (–; *Sb*;*j*)	• The computed alignment score of each pair of elements *V*(*a*, *b*) is computed by matching the symbol S_a,i_ (of sequence T_i_) with the symbol S_b,j_ (of sequence T_j_), where:
			• *Ω* (.) is the symbol-to-symbol distance;
			• –represents a blank gap in the sequence (e.g. a temporal stretch).
**2**	Final alignment score given by the maximum score among all possible alignments	$V(a,b)=max\left\{ \begin{array}{c} V(a-1,b-1)+\Omega (Sa;i;Sb;j)\\ V(a-1,b)+\Omega (Sa;i;–)\\ V(a,b-1)+\Omega (–;Sb;j) \end{array}\right.$	*• V*(*a*, *b*) final alignment score between subsequence with 1≤ a,b ≤ n in our case having sequences of length n.
			• Ω(S_a,i_, S_b,j_) symbol-to-symbol score defined by Eq 3
**3**	Symbol-to-symbol score	$\Omega \;(S_{a,i},S_{b,j})=\left\{ \begin{array}{c} 2\;*\;c_{b}\;if\;c_{b}\geq 0.5\\ 2\;*\;(c_{b}-0.5)\;if\;c_{b}<0.5\\ 0\;if\;either\;symbols\;are\;gaps \end{array}\right.$	• *Ω*(.) symbol-to-symbol distance
			• S_a,i_ symbol of sequence T_i_
			• S_b,j_ symbol of sequence T_j_
			• *c*_*b*_ similarity between two symbols; it is defined differently according to the feature we are basing the cluster analysis on: trajectories (Eq 4) or actions (Eq 5)
**4**	Symbol-to-symbol similarity for trajectories	$c_{b}(i,j)=e^{-\sqrt{{\left(x_{i}-x_{j}\right)}^{2}+{\left(y_{i}-y_{j}\right)}^{2}}}$	• Negative exponential of the Euclidian distance between pairs of coordinates in time (*x*_*i*_*−y*_*i*_*; x*_*j*_*−y*_*j*_) dog coordinates at time *i* and time *j* inside the pen
**5**	Symbol-to-symbol similarity for actions	$c_{b}=\sqrt{det(M^{T}M)}$	• *c*_*b*_ is the square root of the determinant (det) of a Gramian matrix (*M*^*T*^*M*)^a^
			• M is the matrix^b^ obtained by projecting the first k eigenvectors of *L*_*a*,*i*_ (computed using matrix *W*_*a*,*i*_) on the k eigenvectors of *L*_*b*,*j*_, with *L* (Laplace operator) defined by Eq6
**6**	Laplace operator	$L=I-D^{-\frac{1}{2}}WD^{-\frac{1}{2}}$	• *D* is a diagonal matrix^c^ such as *D*_*i*,*j*_ = ∑_*j*_ *w*_*i*,*j*_
			• I is the identity matrix^*c*^
			• *L*_*a*,*i*_ and *L*_*b*,*j*_ are calculated with *W*_*a*,*i*_ and *W*_*b*,*j*_ being the matrices representing the graph of symbols S_a,i_, and S_b,j_ respectively^b^
			• Given two Laplace operators, the graph similarity is computed through measuring the angles between the eigenvectors of Laplacians graph discarding low frequency components and retaining the main structure

^*a*^ A Gramian determinant is used to calculate the volume of a parallelotope (i.e. generalisation of a parallelepiped in higher dimensions). In this case, the parallelotope is created by the divergence between two graph structures. Thus a Gramian matrix is defined by G = M^T^M where M is a real matrix^*b*^ and the vectors are elements of an Euclidean space.

^*b*^In computer science a matrix can be used to represent a finite graph. The elements of the matrix indicate whether pairs of vertices are adjacent or not in the graph.

^*c*^ A matrix is a rectangular array of numbers, symbols, or expressions, arranged in rows and columns. A diagonal matrix is when all entries outside the main diagonal are zero. The identity matrix is a type of diagonal matrix where all the elements on the main diagonal are equal to 1 and all other elements are equal to zero.

The individual alignment scores are calculated on the basis of an arbitrary scoring system (Eq 3, Table 1). The advantage of global alignment over conventional time series analysis algorithms such as Dynamic Time Warping (DTW) is the possibility of controlling the gap score in the alignment process. In our problem, we assigned +2 for two perfectly matching symbols (e.g. equals), -1 to mismatching symbols and 0 to symbols aligning with a gap. This score adopts a low gap penalty thus weakly penalising sequences recorded at different frame rate. This property in our case allows to obtain a significant similarity score even if the dogs are performing similar actions but at different speed. Detailed comparison between the two algorithms in time series analysis can be found in [40].

Eventually the NW algorithm uses the results from the sub-sequences to reconstruct the solution of the original problem (i.e. full length sequence).

The global alignment algorithm uses these scores for the alignment of the sequences. This programme can be used for the cluster analysis of either the trajectories or the behaviour (i.e. body posture).

The trajectories are described as temporal sequences of the coordinates (*x*_*i*_*; y*_*i*_) of the dog (i.e. position of the barycentre of the dog inside the pen at time i with i = 1…T where T is the temporal length in frames of the trajectory). Thus, the scoring will be computed according to Eq 4 (Table 1). This analysis allows categorising the dog behaviour according to the type of movement and position in the pen (e.g. stops in front of pen, walks along one side of the pen, moves in circle)

**Fig 4:**
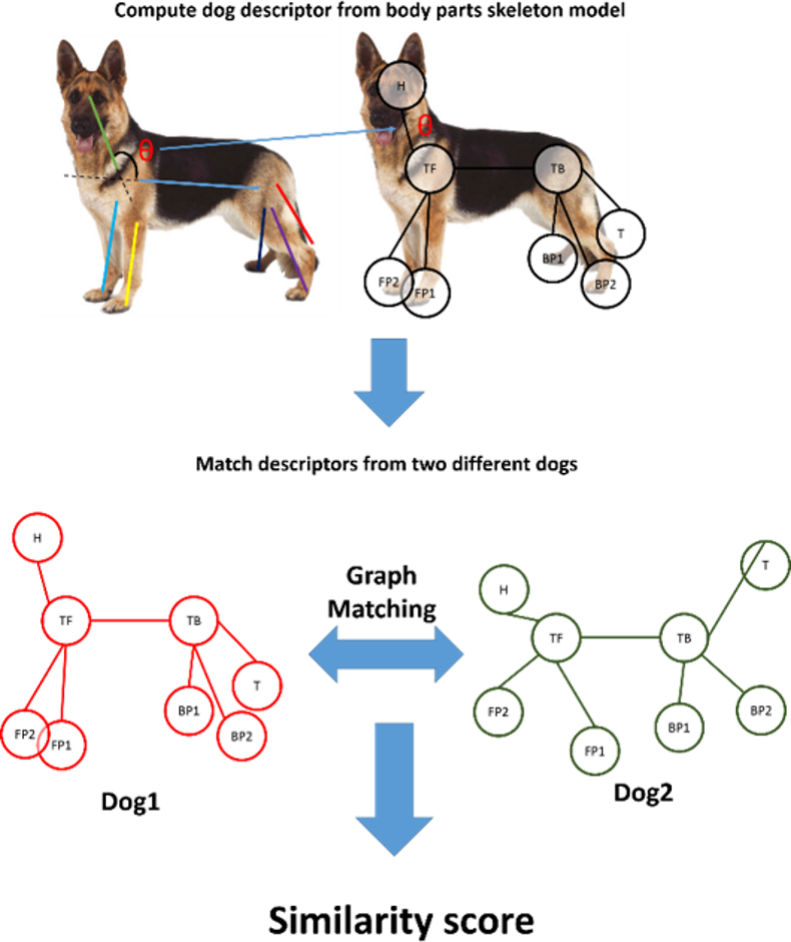
Comparison between different frames of the dog skeleton. For each frame a descriptor score for the dog skeleton is computed (top). Then, all the descriptors (from the same or different dogs) are compared and matched. The NW algorithm creates the similarity scores and aligns the segments.

The clustering of the body postures, instead, is more complex and it is performed by computing a descriptor score from the skeleton model of the dog. In particular, the whole body parts are used to describe the dog actions. Precisely, given the extracted body parts, those are arranged frame by frame on a graph where the vertex are the body parts junction and the edges encodes the angular distance with relation to these junctions as shown in Fig 4.

The symbol-to-symbol similarity *c*_*b*_ of Eq 3 is specified for the action analysis task as the divergence between body parts graphs from different sequences (Eq 5 Table 1). This algorithm uses the Laplace operator (or Laplacian) [41], which is able to capture the structure of the graph and it is hence suitable for graph comparison even in the presence of measurement noise such the one that typically occurs with the dog paws (Eq 6 Table 1).

Once all matches are computed, the NW algorithm uses this symbol-to-symbol measure between frames information to assign the similarity scores and process the segment alignment, as described previously using NW score tables.

This algorithm uses the patterns of movement of all body parts in time, and does not rely on semantic labelling (e.g. pre-defined postures). This model measures all angular variations occurring between different body parts allowing a very detailed analysis that could not be performed manually by a person. Thus, each pattern of movement is mathematically defined by a collection of temporal sequences with specific skeletal angles. Differently from the trajectory analysis, here only the skeleton movement in time was implemented in the model, and not the position in the pen, since not relevant to characterise the action.

**Fig 5:**
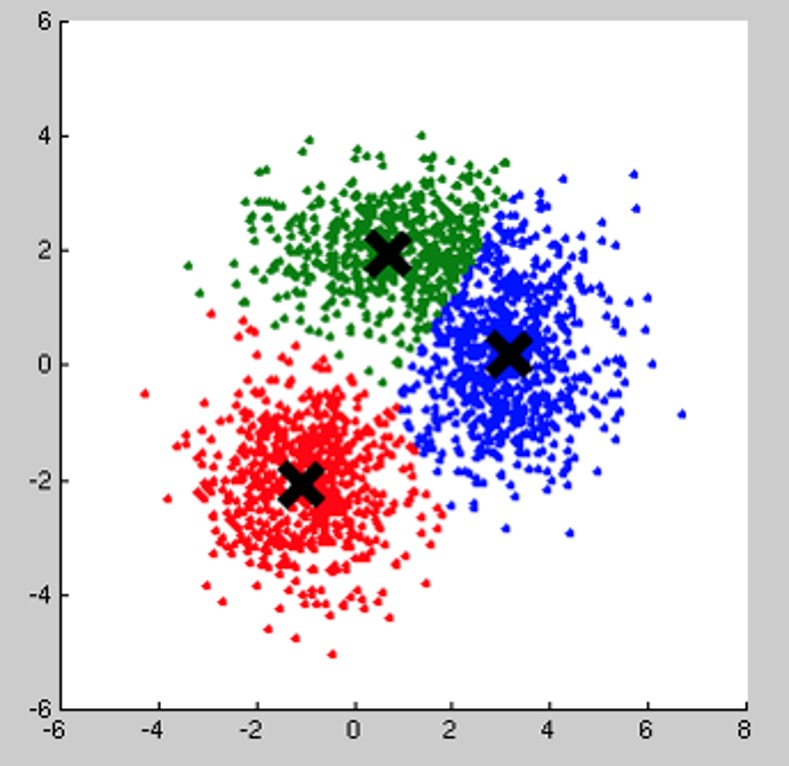
Clustering analysis. Each dot represents a video segment. Distance between dots is computed by the alignment algorithm either on coordinates (i.e. trajectories) or body parts (i.e. actions). In this example, the system created three clusters (green, blue and red clouds) where the centroid (black cross) is the most representative sequence of the cluster. The more the sequences are close together the more the alignment produces a high similarity score. Sequences distant from the centroid are less similar than the computed action prototype.

The results from the alignment algorithm (i.e. the similarity among different sequences) are then processed for the actual clustering. Given the segments similarity matrix, the algorithm (Kmedoids clustering [42]) partitions the set of sequences in cluster depending on the desired feature, either the dog coordinates or its body parts. The effect of the grouping is that similar behavioural patterns fall in the same group, even if performed at different times (Fig 5).

The B.A.R.K. software is able to create an output on the basis of default settings. However after a visual inspection of the clustering, the researcher is able to change the time interval in which the video is partitioned and the number of clusters to create (see S1 Appendix, section 5). If the system creates, for example, 5 clusters, but two of them are not meaningfully different for the research purposes, it is possible to reduce the number of clusters to merge even more similar segments, and vice versa it is possible to increase the number of clusters if a more fine analysis is required. From the output it will be possible to visually inspect all segments of videos in their clusters, the video sequences are ordered according to the distance from the cluster centroid. At the top of the list the sequences are visually more similar. As a rule of thumb, the number of clusters could be increased if video sequences at the bottom of the list are completely different from their cluster centroid consequently a finer clustering is needed.

### Validation procedure

The accuracy of the B.A.R.K. system in identifying and recording changes in behaviour was assessed.

The software was trained to identify and label four basic behaviours: stand on four legs, locomotion, sit and lie on the floor.

#### Accuracy in detecting dog body postures

The first step was to evaluate the accuracy of B.A.R.K. to correctly define dog body postures in different scenarios (i.e. indoor/outdoor).

Two different real scenarios (i.e. laboratory and shelter) were considered with more or less controlled lighting condition. Tests were performed on randomly selected sequences, including different breed-types from both recording sessions, for a total of 30 min of video per trial, 10 frames per second. Trial 3 contained sequences acquired with constant artificial lighting conditions (laboratory recording). Conversely, Trial 1 and Trial 2 were characterised by natural light in an outdoor pen (shelter recording) which could be constant or more unstable throughout the video clips. The total number of frames considered for classification was 8500 for Trial 1, 7500 for Trial 2 and 6000 for Trial 3; in all the frames the dogs were visible and awake. Ten percent of these frames, randomly chosen for every test trial, were manually annotated and used for quantitative results. In machine learning, the manual annotation of the researcher is called Ground Truth, GT. The accuracy scores were computed using the Percentage of Correctly estimated body Parts (PCP), a well-established performance measure for human body posture estimation from 3D sensors [43].

#### Behavioural analysis

The validation procedure included a comparison between the parameters estimated by inference (i.e. by the B.A.R.K.) and the manual annotation (GT) of 13 video clips by a blind experimenter. The videos were sampled from both the lab and kennel situation (3 dogs were in one video-clip, 1 dog was in 4 clips and 1 dog was in 6 clips according to image quality), 1-hour duration for a total of 25K frames. For each sample frame, the experimenter was asked to label the posture of the dog (choosing from stand, sit, locomotion and lie, all mutually exclusive) and to determine the duration of each behaviour using the Observer XT software (Noldus Innovation and Technology, The Netherlands).

Correspondence between the two recording methods was assessed following three steps:

1. A per-frame classification, considering all frames independently, by associating the B.A.R.K. output and the GT in a confusion matrix. Confusion matrices are commonly used in machine learning science: if a classifier has been trained to distinguish between different classes, the confusion matrix allows a visualization of the performance of the algorithm. The predicted classes (by the classifier) are in the rows and the actual classes in the columns (or viceversa) making it easy to spot an error (i.e. confusion) since this will be located outside the diagonal (correct guesses will be on the diagonal).
2. A temporal analysis by visually inspecting the system performance in annotating the amount of time the dog spends in a specific pose.
3. A correlation analysis to assess the agreement between the two sets of data scored using the software or the GT (Spearman’s rho test. Alpha = 0.05 for all comparisons).

#### Clustering analysis

In the result section, an example of clustering analysis is presented, based on the categorization of behaviour of one dog video recorded for 50 seconds. An ethologist visually inspected the cluster analysis output in order to describe the patterns of behaviour characterising each cluster and to label them.

## Results

The B.A.R.K. system was tested against different experimental settings to evaluate the software performances in different tasks.

### Accuracy in detecting dog body postures

**Fig 6:**
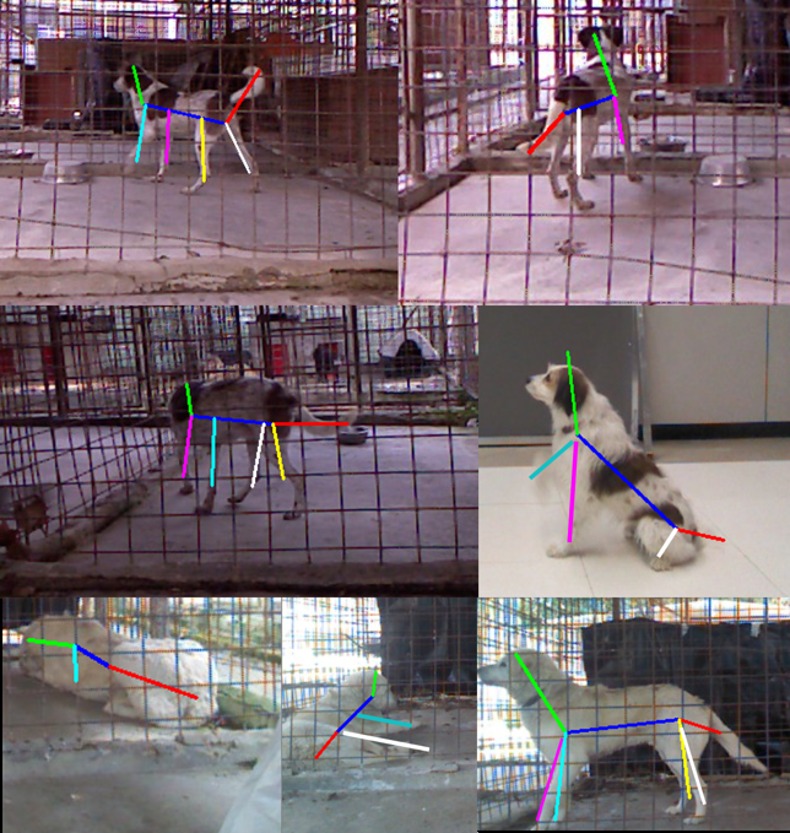
Qualitative Examples of Body part detection. Each image shows an example of the extracted dog body part in different conditions. Different line colours correspond to different body parts.

The first evaluation campaign measured the accuracy of the B.A.R.K. system in correctly detecting the dog behaviour according to the SSVM-based algorithm previously described. The body postures chosen from the output of the three SSVM classifiers (one for each posture i.e. sit, lie, and stand) that has the best score. Quantitative results (Table 2) show that the overall accuracy of the software in identifying the correct posture improves with increasing number of training examples. Examples of qualitative results (i.e. how accurate is the overlap between the RGB image of the dog’s posture and the extracted skeleton at visual inspection) are shown in Fig 6.

**Table 2:** Variation in the Percentage of Correctly estimated body Parts (PCP) with increasing training size and different light conditions.

	Training Size			
	67	128	228	385
**Trial 1**	65%	72%	77%	80%
**Trial 2**	55%	63%	69%	74%
**Trial 3**	59%	74%	79%	83%

Trial 1 kennel environment with constant/natural light conditions; Trial 2 kennel environment with unstable/natural light conditions; Trial 3 laboratory environment with controlled/artificial lighting conditions.

Observing the results (Table 1) the system is capable to achieve satisfying performances in terms of PCP both in indoor and outdoor situations.

We also compared our system against two state of the art methods, the first specifically designed for humans and based on SSVM [44] and the second designed for dogs but using a single SSVM classifier independently from the labelled postures [35] (Table 3).

**Table 3:** Comparative results of our PCP against the state of the art methods. Bold values are the best.

	Our	(Chen et al 2011) [44]	(Pistocchi et al 2014)[35]
**Trial 1**	**80%**	50%	70%
**Trial 2**	**74%**	48%	67%
**Trial 3**	**83%**	53%	78%

By observing the results in Table 3, our proposal performs better than the two competitors. The improvement over [44] is mainly due to the adoption of better features and a more sophisticated solution generator in the SSVM inference process. The improvement over our previous proposal in [35] is mainly due to the adoption of three different classifiers capable of generating the most suitable solutions according to the dog posture. Although the classification algorithm is the same, even in this case there is an average improvement of performance.

### Behavioural analysis

The experiment has been carried out testing the capability of labelling correctly the dog’s posture and behaviour.

**Fig 7:**
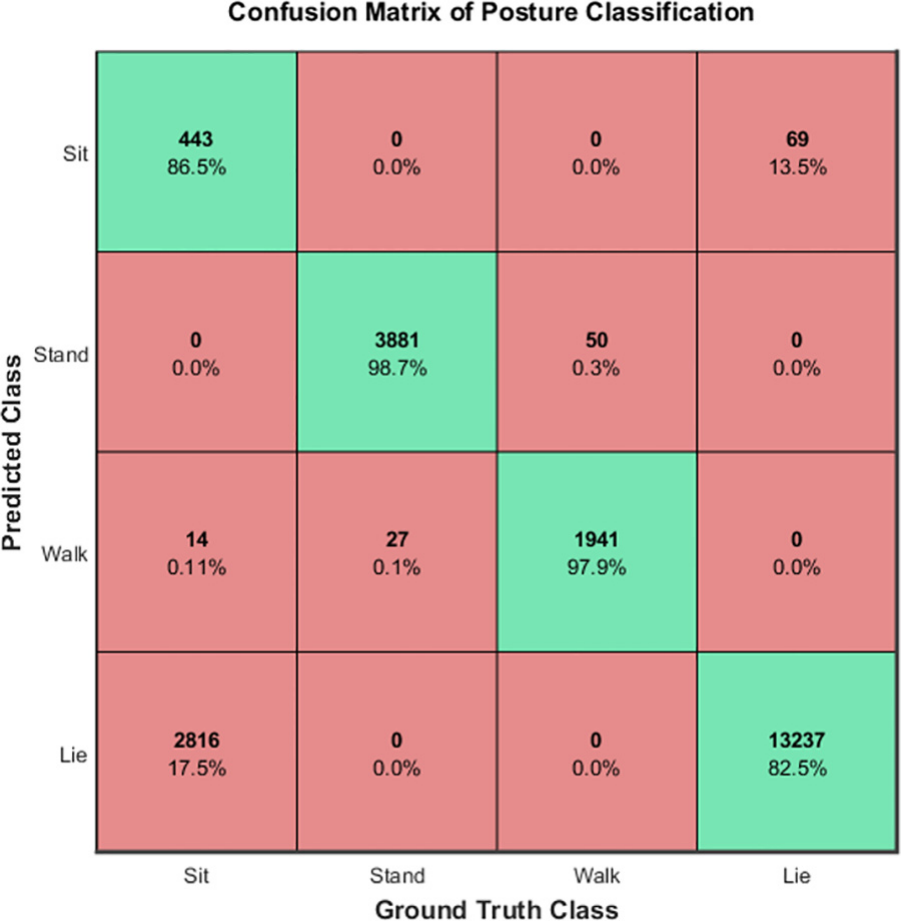
Confusion Matrix of the B.A.R.K. posture classification (predicted class) compared to the manual annotation (Ground Truth class). Numbers and percentages of samples corresponding to the system outputs are reported in the cells. Ideally, values in the green diagonal should aim at 100% accuracy.

**Fig 8:**
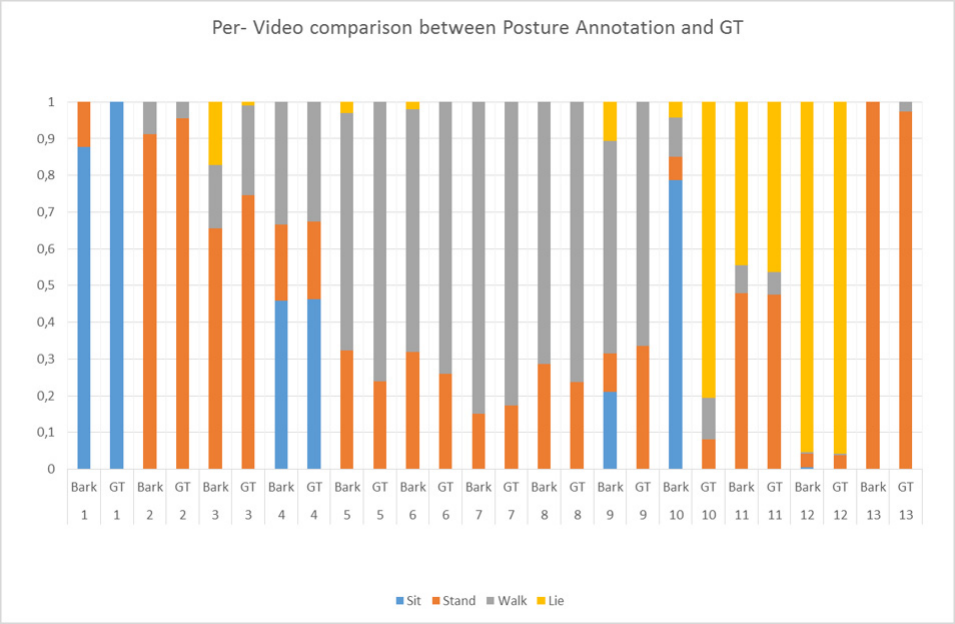
Bar-plot of the percentage of time dogs spent in a specific posture: The y axis is the percentage of the video while the bars graphically depict how the video was scored in terms of behaviours. On the X axis the B.A.R.K. results and the Ground Truth are shown for every video.

1. The first experiment evaluated the classification results per-frame considering all frames independently. Fig 7 shows the confusion matrix of the classification system. Every row corresponds to the B.A.R.K. outputs while columns are the Ground Truth (GT). Cells contain the number and the percentage of samples corresponding to the system outputs while the GT class is identified by the column index. An ideal classification with no errors should be a diagonal matrix with 100% on the diagonal. Observing the confusion matrix it is possible to grasp the capability of the system of correctly identifying the right dog posture and behaviour in most of the cases. Stand and walk (locomotor behaviour) classes have an average accuracy of > 95% while sit and lie classes of 86% and 82% respectively. Moreover, by observing sit and lie rows, it is evident that most of the errors involve confusing these two classes. This behaviour is a consequence of an inner ambiguity between sit and lie where the observer itself should take a decision when the dog posture is on the boundary between the two classes, e.g. the dog is sitting but still very close to the floor.
2. In conjunction with the per-frame analysis, a temporal analysis has been carried out evaluating the system performance in terms of annotating the amount of time the dog spends in a specific pose. Fig 8 presents a bar-plot where for every video its temporal length is partitioned according to the detected behaviour. Observing the plot it is clear how the B.A.R.K. system is capable of computing a solution close to the actual values. The only marked exception being clip number 10 where there is confusion between the classification of sitting versus lying behaviour. On a closer inspection, the dog in the clip had for most of the time the lower body (legs) on the ground in a lying position but the head and torso held high, creating an ambivalent posture.
3. Finally, when comparing the two type of scoring (automated and manual) of the 13 clips, the Spearman test showed a high correlation between scoring systems for most variables (Table 4). The lowest correlation was associated with sit behaviour, confirming that classification mismatch already spotted through visual inspection (Fig 8).

**Table 4:** Correlation between the manual annotation and automated scoring of behaviour using B.A.R.K. Spearman’s rho and p-values are presented.

	Sit	Stand	Locomotion	Lie
**rho**	0.59	0.92	0.95	0.81
**p-value**	0.04	<0.0001	<0.0001	0.001

### Clustering analysis

**Fig 9:**
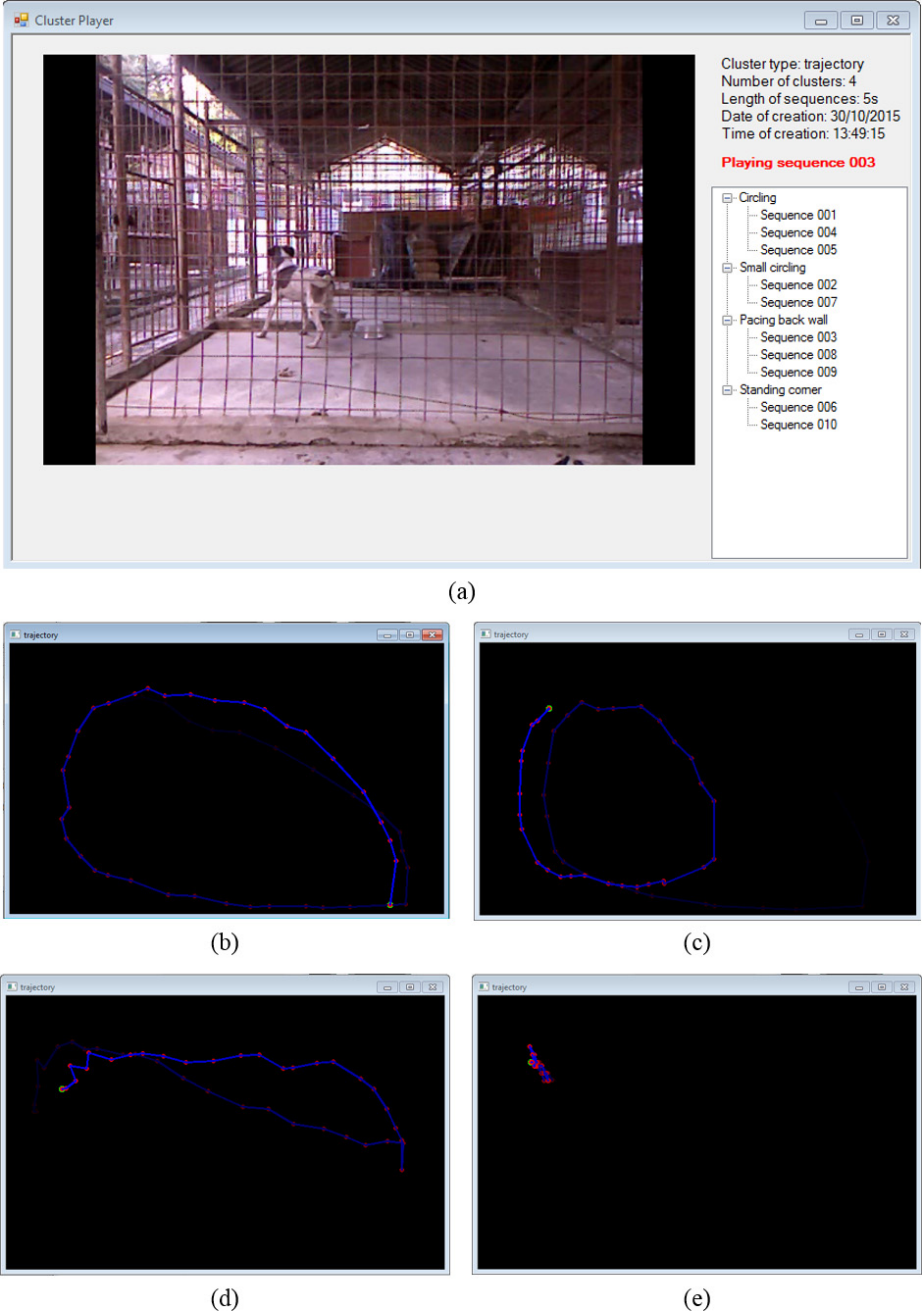
**Example of automated cluster analysis** (a) dialog window showing the cluster analysis results. On the right menu the 5sec sequences are grouped into 4 clusters that were manually renamed after visualising them all (i.e. double-click on the sequence to play). The different dog’s trajectory performed in the sequences in cluster 1 (b), cluster 2 (c), cluster 3 (d) and cluster 4 (e) give an idea of how the B.A.R.K. automatically groups different patterns of behaviour expressed by the dog in the clip.

The visual inspection of the clips grouped by the automated cluster analysis gave the following results:

Video 1: Ten 5sec sequences were created and grouped in 4 clusters (Fig 9A)

- Cluster 1(3 sequences) the dog circles along the perimeter of the pen (Fig 9B and S1 Clip)
- Cluster 2 (2 sequences) the dog circles on the front half of the pen (Fig 9C and S2 Clip)
- Cluster 3 (3 sequences) the dogs paces along one side of the pen (Fig 9D and S3 Clip)
- Cluster 4 (2 sequences) the dog stands in one corner of the pen (Fig 9E and S4 Clip)

## Discussion

In this study, we introduced and validated a new 3D system able to detect and analyse dogs’ body parts and recognise some major behavioural patterns in time and space.

Behavioural measures are honest signals, reflecting how the animal is coping with its environment [1]. Posture and locomotor activity (e.g. time spent moving, standing, sitting and resting) have been used previously as indicators of dog welfare, especially when monitoring confined animals [18,27,30,32,45]. Spangenberg and others [46] for example showed that granting laboratory dog’s access to outdoor runs increased their activity levels, which was considered beneficial for their welfare. Beerda and colleagues [29] found that social and spatial restriction led to a decrease in activity, excessive grooming and vocalisations, alterations in exploratory and locomotor behaviour. In Dalla Villa et al. [18] postures and activity were affected by the confinement condition. Dogs walked and trotted considerably less (86% and 95% less respectively) and were lying much longer when housed in smaller pair kennels compared to larger group ones. Similar results were found by [27] and by [47] when comparing single-housed versus group-housed dogs. Recently, Clarke and Fraser [22] developed an automated monitoring system to specifically detect resting behaviour (especially head-down posture) in shelter dogs. Shelter dogs can be subjected to many stressors which may affect their ability to rest for an adequate amount of time. Lack of rest is therefore another useful indicator of welfare impairment.

An animal’s position within the pen has been a useful marker in welfare studies as it correlates with the likelihood that an animal will be adopted or be more desirable. Wells and Hepper [32] for example showed how some elements of the environment (i.e. position of the bed and social interactions) increase the likelihood of a dog to stand at the front of the pen, more visible to the visitors and thus more desirable for purchase. Again, Wells et al [30] reported that dogs housed in the shelter for over five years spent more of their time at the back of their kennels, more time resting, and less time barking than dogs housed in the shelter for shorter periods of time. This attitude made the animals less desirable and thus less likely to be adopted.

The B.A.R.K. software is still a prototype and several improvements could be made. However, being able to detect and analyse these behaviours in an automated way could provide a great advantage in animal welfare science.

For this pilot experiment, the learning machine was trained to recognise and label four main postures/behaviours, but future work and training may allow detection of novel postures (e.g. rearing on hind legs). Future work may also improve the algorithms used to detect more fine behaviours such as the intensity and frequency of tail wags, vocalisations (by adding sound), or to record multiple subjects in the same environment analysing their social interactions [12]. Finally, environmental features could be added to the background as an integral part of the analysis (e.g. dog stands on top of a piece of furniture or hides under it). Indeed, the next step could be to train the software to recognise and differentiate between multiple individuals in one pen and their interaction.

The algorithms developed for this study and applied to the dogs’ body-parts can be adapted and generalised to other quadrupeds, broadening the fields of application to laboratory and zoo animals. At the moment, the software was tested on five dogs with different coat colour and length and B.A.R.K. seems to generalise well. The software should not be affected by differences in the shape or size of dogs because the skeleton is created from the blob of each individual animal. However, the system might suffer poor accuracy whenever there are occlusion problems, especially of the legs, for example dogs with very long hair or very short legs (e.g. dachshunds). Further testing should address these issues.

A major innovation of B.A.R.K. is the capability to train the software to recognise and label specific behaviours without being restricted by this training as a compulsory step. The system is able to self-generate and recognise patterns of behaviour in space and time, with no a-priori input or outline of a species-specific ethogram. The B.A.R.K. computes the variation in the angles between connected body segments and their position in three-dimensional space. On the basis of mathematical algorithms, similar patterns of behaviours can be automatically grouped together, highlighting any deviations from normal or baseline. In the example reported in the result section, the B.A.R.K. software grouped those sequences where the dog was circling along the fence of the pen, or circling in a tighter circle in half the space (Fig 9B and 9C) separately from other walking patterns (e.g. pacing on a line along one side of the pen, Fig 9D) or other behaviours as standing still in one corner (Fig 9E). It is important to point out that, although the machine was trained to detect few species-specific postures and label them, the cluster analysis was not considering these constraints. In the future, a refinement of this tool could allow the set-up of early warning systems able to detect the insurgence of undesirable behaviours such as stereotypies or intense aggressive reactions by the identification of specific predictors [6]. Deviation from ‘normal’ activity levels may be an indicator of ‘restlessness’, hyperactivity or the development of stereotypical behaviours (predicted by unusually high activity) or of apathy and depression (predicted by unusually low activity). Stereotypical behaviours are repetitive and unvarying behavioural patterns and are often considered an indicator of poor welfare [48]. However, the prevalence of this behaviour in a shelter population might not be very high (e.g. 0.3% of population [18], less than 5% in [27], 10% in [47]). A system recording and analysing data continuously can detect rare or unusual patterns of behaviour that could be missed when applying common sampling techniques.

Automated behavioural analysis has the advantage of a computer algorithm that ensures high accuracy and standardisation as well as real-time feedback. Long-term recording is also a great advantage, allowing a large volume of data storage and analysis with minimal effort, saving the high cost of human labour. In line with previous findings [49,50], the automated video analysis was highly correlated to human observations (i.e. Ground Truth). This means that after training, the software was detecting meaningful dog behaviours in a reliable way. A lower correlation was found for one posture in particular (Table 4). A visual screening of the clips detected one dog that held an ambiguous posture for most of the video thus leading to a classification mismatch. Additional training of the learning machine (through a larger and more variable set of data) could reduce this error improving the overall agreement levels.

The use of only one Kinect sensor (instead of multiple cameras as proposed in other 3D studies e.g. [12,51]) positioned at eye level (instead of a top view) makes the acquisition of the depth images easy and affordable and can be adapted to almost any experimental or clinical trials (as long as it is a controlled environment). The Kinect camera has built in infrared sensor so it works well in poor or absent lighting. Conversely, it suffers from high exposure (e.g. direct sunlight); in such cases the quality of the image can be compromised and the software could be less effective in the segmentation process and in identifying the dog skeleton (Table 2). This problem may be partially solved when using more expensive stereo cameras, or when working in artificially lit environments rather than open air ones.

The outburst of new video technology and computer image processing, led to promising results in human surveillance, where people behaving differently or suspiciously from the rest of the crowd could be automatically detected and the information reported to security [52]. Similarly, this tool could become important to detect risk factors that potentially compromise a dogs’ welfare e.g. a prolonged state of apathy characterised by unusual motionless or other clinical problems. Other potential applications include automatic recording of a dogs’ behaviour in a clinic, in a home environment when unsupervised or in any other experimental setting, retrieving real-time feedback of the dog behaviour in pre-defined situations.

## Conclusion

This is, to our knowledge, the first time that a 3D software is developed to automatically record the behaviour of the domestic dog in a kennel environment using a single depth camera. As a prototype, this tool has a high margin for improvement and high potential. In the age of technology, this is a necessary step toward a major turnover in the way that behaviour in large quadrupeds is analysed, saving time and resources and increasing data standardisation, accuracy and repeatability.

## Supporting Information

S1 Appendix B.A.R.K. software overview Additional information on the user’s interface of the software and details on it main functionalities are provided.
(DOCX)

S1 Clip Video-clip example. Exemplificative video sequence (5sec) showing the behaviour characterising Cluster 1 (also represented by Fig 9B)
(WMV)

S2 Clip Video-clip example. Exemplificative video sequence (5sec) showing the behaviour characterising Cluster 2 (also represented by Fig 9C)
(WMV)

S3 Clip Video-clip example. Exemplificative video sequence (5sec) showing the behaviour characterising Cluster 3 (also represented by Fig 9D)
(WMV)

S4 Clip Video-clip example. Exemplificative video sequence (5sec) showing the behaviour characterising Cluster 4 (also represented by Fig 9E)
(WMV)

S1 Supporting Article Conference proceeding Pistocchi et al. 2014. Article describing the algorithms behind the early developmental phases of the software. Corresponds to reference [35].
(PDF)
