# Supplementary material for: Quick, Accurate, Smart: 3D Computer Vision Technology Helps Assessing Confined Animals’ Behaviour
Source: PLoS One. 2016 Jul 14;11(7):e0158748. doi: 10.1371/journal.pone.0158748 (PMC4944961; doi:10.1371/journal.pone.0158748)
Supplement: S1 Appendix — Additional information on the user’s interface of the software and details on it main functionalities are provided. (DOCX) [file pone.0158748.s001.docx]

**S1 Appendix: B.A.R.K. software overview**

This supplementary material has the purpose of giving the reader an overview of the user interface and functionalities of the B.A.R.K. software. It is not meant to be an exhaustive user manual so some steps or features (irrelevant to the study) have been omitted for the sake of brevity.

Below, a schematic representation of the steps performed by the software from the segmentation of the animal to the generation of dog body-parts solutions as described in the main text.

**
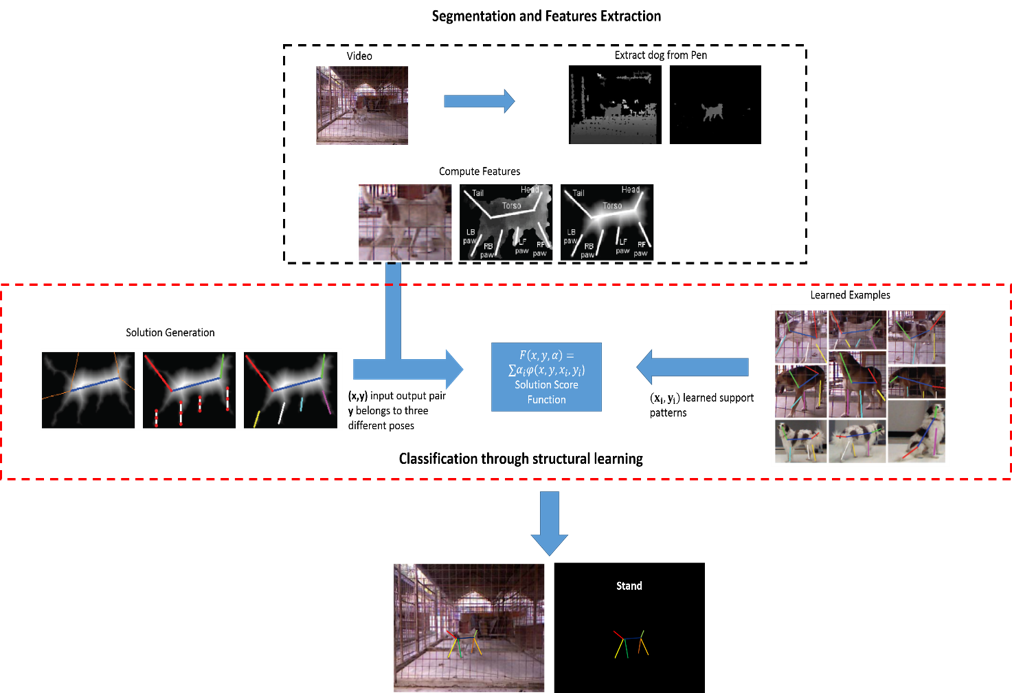
**

d)

c)

b)

a)

**Software overview** a) A frame image from the 3D video is processed to isolate the dog from the background (i.e. segmentation) by working on the depth image. b) The dog body-parts solution features are represented over a depth image and a *distance transform* image. c) The dog body-parts detection and training of learned examples of postures allow the maximisation of the solution score function to match input-output pairs. d) The structural classifier generates high score solutions (i.e. posture labelling).

1. ***Video acquisition and upload***

On launching the program, several actions are available.

On the right side of the user interface window (Figure 1), it is possible to add information about the subject such as name, age, breed, sex and category (e.g. treatment). The *Save dog information* button will activate after any changes are made.


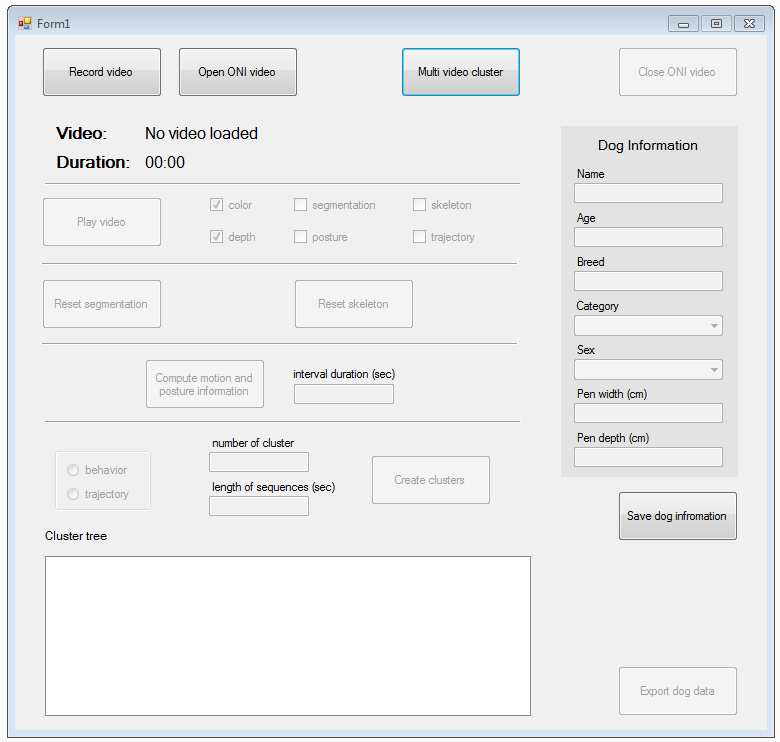


**Figure 1** shows the user interface when launching the B.A.R.K.

The researcher can

- Start a real-time video acquisition: connect a Kinect sensor to the computer, set it up and click on *Record video* (Figure 1)*.* A new window will appear with the immages that are beeing acquired.
- Upload a video: click on *Open ONI video* (Figure 1)*,* select an .ONI (OpenNI) file from the dialog window.

1. ***Video player***

After the video has been acquired or uploaded, it is possible to watch it by pressing the *Play video* button (Figure 1). A new window will appear running the video (Figure 2) and a legend with the keys needed to play, stop, change speed etc. (e.g. *spacebar* to play/pause video; *backspace* to restart the video)

When playing a video, the following options/features can be selected (some will be active only after specific steps have been performed):

- Colour: play the video as it was acquired by the Kinect sensor (Figure 2a)
- Depth: show the depth map of the video (Figure 2a)
- Posture: the label of the posture will appear on the top-left corner of the window. Labels change as the posture of the dog changes (active only after detection of dog body parts see section 4) (Figure 2a)
- Segmentation: shows the blob of the subject isolated from the background (active only after segmentation, see section 3) (Figure 2b)
- Skeleton: the 7 main dog body-parts segments will overlap the dog blob (active only after detection of dog body parts see section 4) (Figure 2c)
- Trajectory: a window will open with the top view of the movement of the dog in time inside the pen (active only after trajectory calculation see section 4) (Figure 2d)

(a)
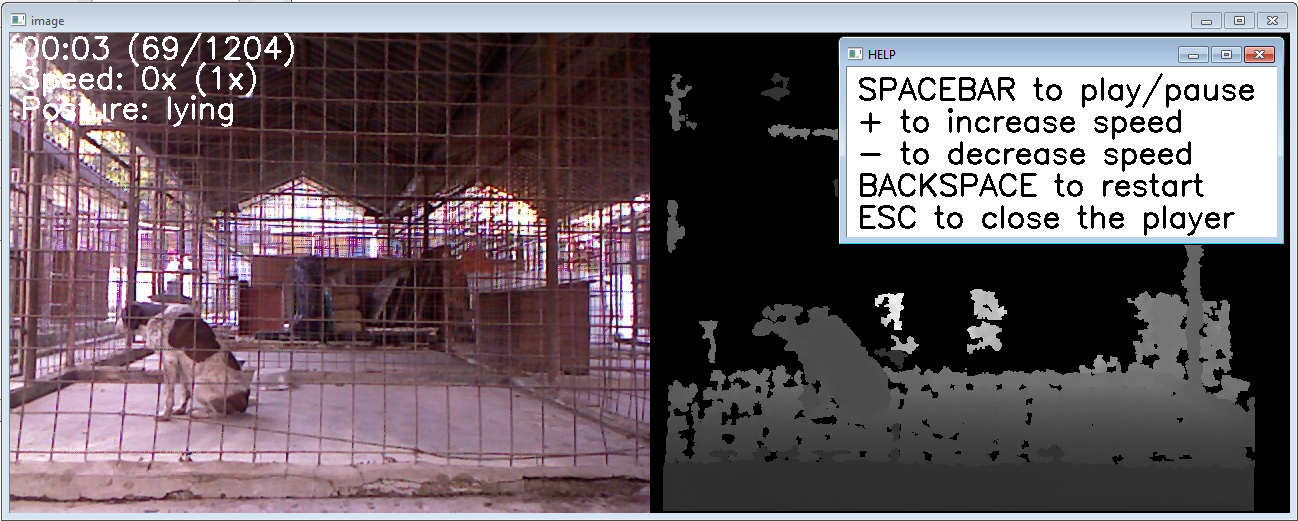


(b)
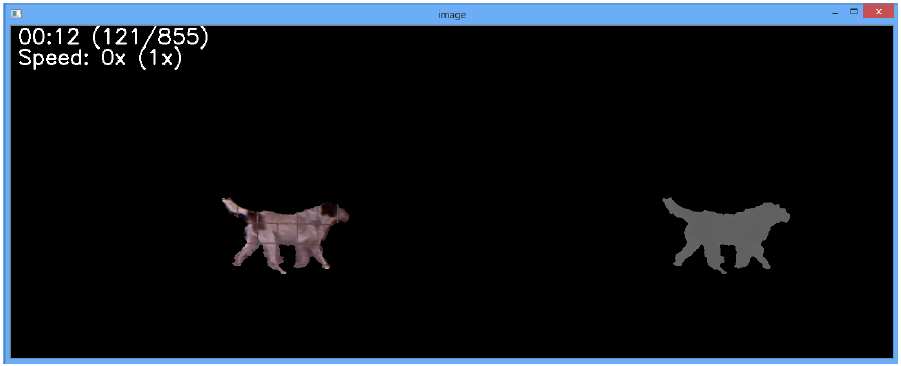


(c)
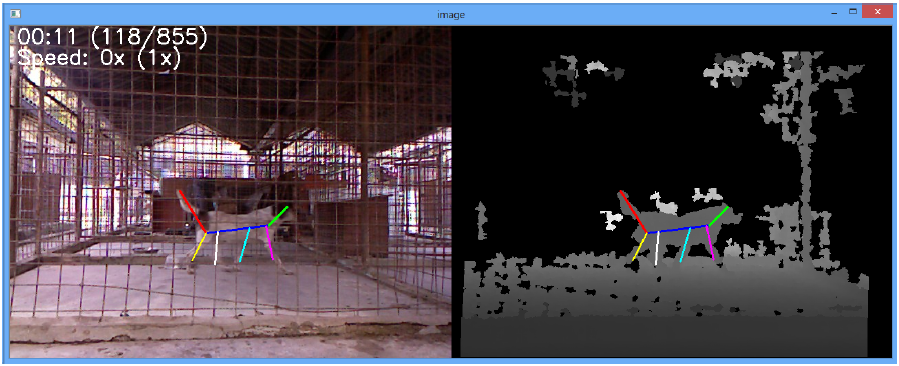


(d)
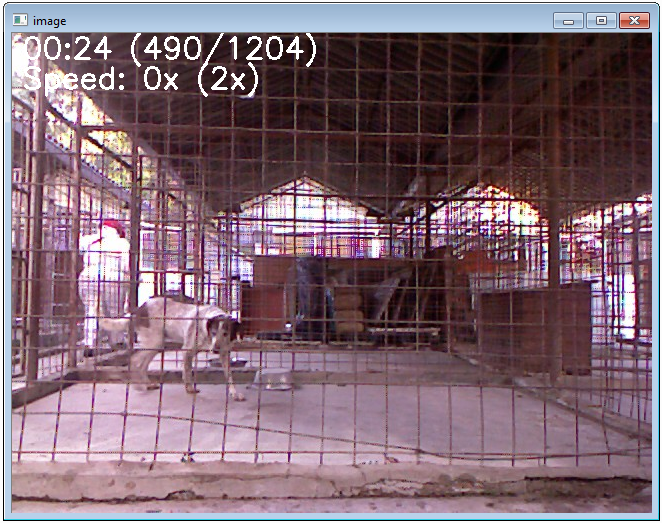

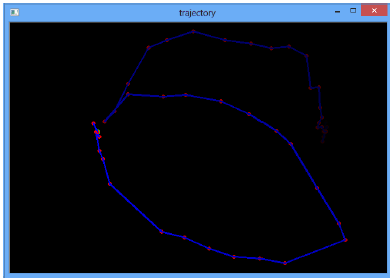


**Figure 2** shows the video played when selecting (a) the colour, posture and depth, (b) the segmentation, (c) the skeleton, (d) or the trajectory option.

1. ***Segmentation***

Before starting any analysis, the subject has to be isolated from the background, and the planes of the pen (i.e. ground/walls) need to be defined, excluding everything that falls outside the area of interest.

To start click the button *Segmentation*. A new window with the video will appear and clear instructions will help through this two-steps procedure.

1. Define planes: select 5 random points of the pen floor from the depth map (Figure 3), and then select 2 points from the left and the right walls. Press the *spacebar* to confirm.
2. Adjust planes: move the planes to better adjust them to the real perimeters of the pen by comparing them to the colour image (Figure 4).


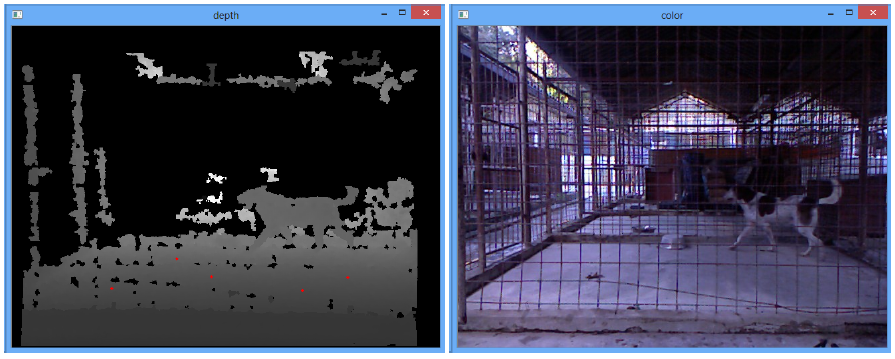


**Figure 3** shows one of the steps to follow when doing the segmentation (i.e. define planes).


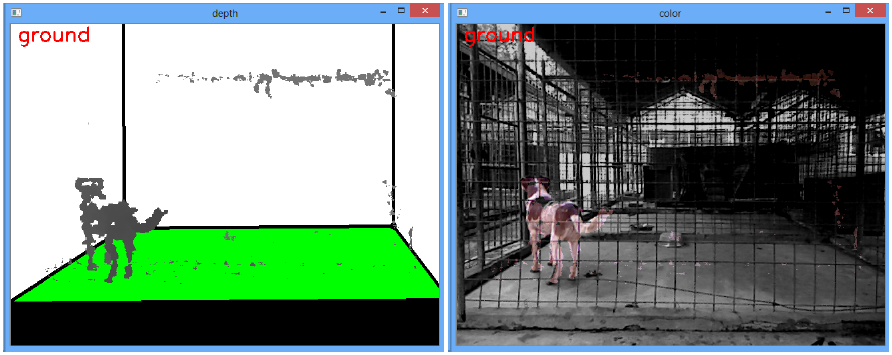


**Figure 4** shows the projections of the planes. By selecting one of the 5 planes (i.e. ground, vertical-left, vertical-right, vertical-rear and vertical-front) it is possible to move them to match as much as possible reality. The selected plane is coloured in green (this example the ground is highlighted) and can be moved up or down by pressing the *+* or *–* keys on the keypad.

The segmentation will be saved for that specific video. Therefore, this procedure needs to be calculated only once. However, it is possible to re-calculate it by pressing the *Reset segmentation* button.

1. ***Computing the dog body-parts and trajectories***

This phase is automatic, just click on the *Calc skeleton* button (Figure 1) to start the procedure. This could take a few minutes, depending on the video length.

It is possible to delete this procedure by clicking on the *Reset skeleton* button.

1. ***Clustering***

This feature will allow to automatically group sections of video(s) into groups according to their similarities based on the behaviour (i.e. calculated according to the movement of the skeleton) or on the trajectories (i.e. movement of the subject in space and time).

To run the clustering it is necessary to define

- The type of clustering (i.e. based on behaviour or on trajectories)
- The ideal number of clusters
- Length of video sequence (seconds)

Press *Create cluster* to run the analysis. If the output is not satisfactory, these parameters can be changed and the analysis re-run.

A cluster tree will appear with all the groups and all the video sequence allocated to each group (Figure 5). The individual sequences can be played in the dialog window by double-clicking on the sequence name in the cluster tree.

These sequences or group can be modified manually by dragging and dropping. Groups can be renamed by right-click and *rename*.


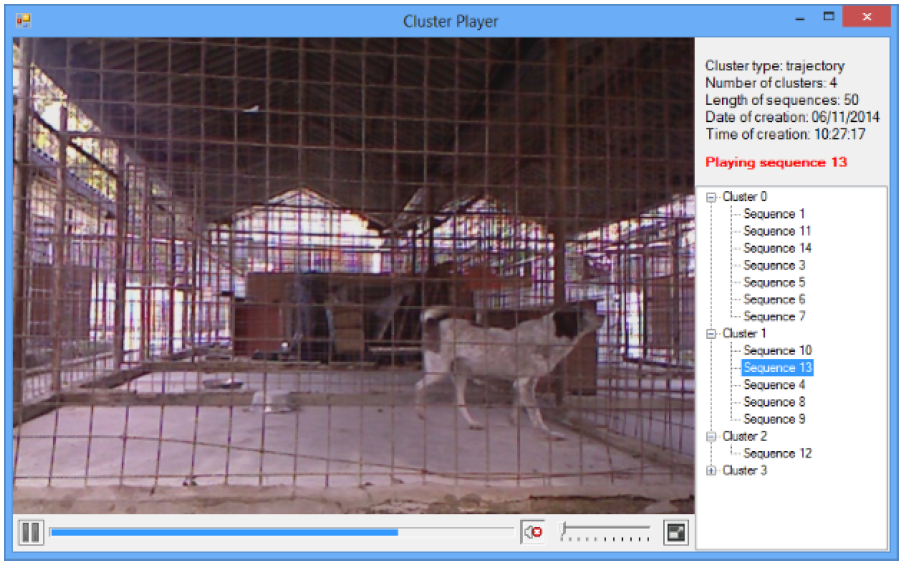


**Figure 5** shows the cluster tree output. To inspect the clustering it is possible to view the videos by selecting the correspondent sequence from the tree. The sequences that appear first, at the top of each group, represent the centroid of the cluster group. If the sequences at the bottom of the list are less similar to the top ones, i.e. if by a visual inspection these appear very different from the top ones, increasing the number of cluster groups may help creating more homogenous clustering.

The B.A.R.K. allows the clustering of multiple videos of the same or different dogs.

Click on *MultiVideo cluster* to start the procedure. A new dialog window will appear allowing the uploading of multiple videos (Figure 6). Only videos that have been already processed individually can be uploaded (i.e. segmentation and calculation of skeleton and trajectories).

When all videos have been uploaded, press *Create cluster* to run the analysis and then follow the steps described above for the basic clustering.


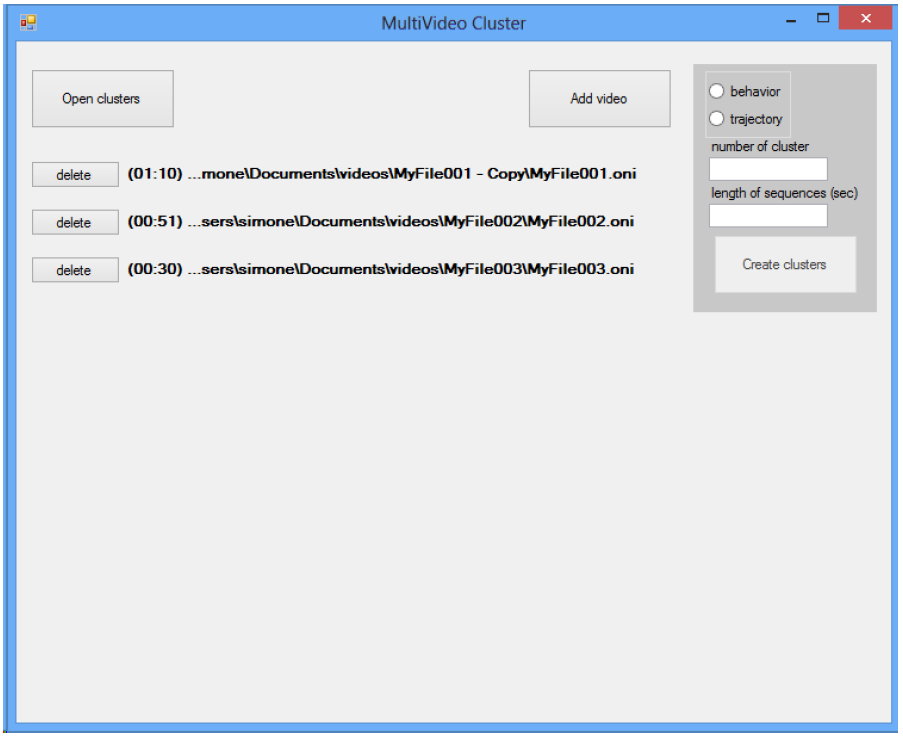


**Figure 6** shows the dialog window for multi video cluster

1. ***Export analysis***

Data can be exported in .cvs format. Click on the *Export dog data* (Figure 1) button to automatically generate a file including:

- Dog information (name, age etc.)
- Length of video
- Duration of behaviours (i.e. sit, stand, lie)
- Type of cluster and duration of cluster groups

The activity level of the dog (in term of time spent in each virtual quadrant of the pen and in meters travelled) can also be calculated automatically. The floor of the pen is automatically divided into nine squares, and the time spent by the dog in each square is computed for the whole duration of the clip or for pre-set time intervals defined by the researcher. The output is an image similar to that presented in Figure 7.


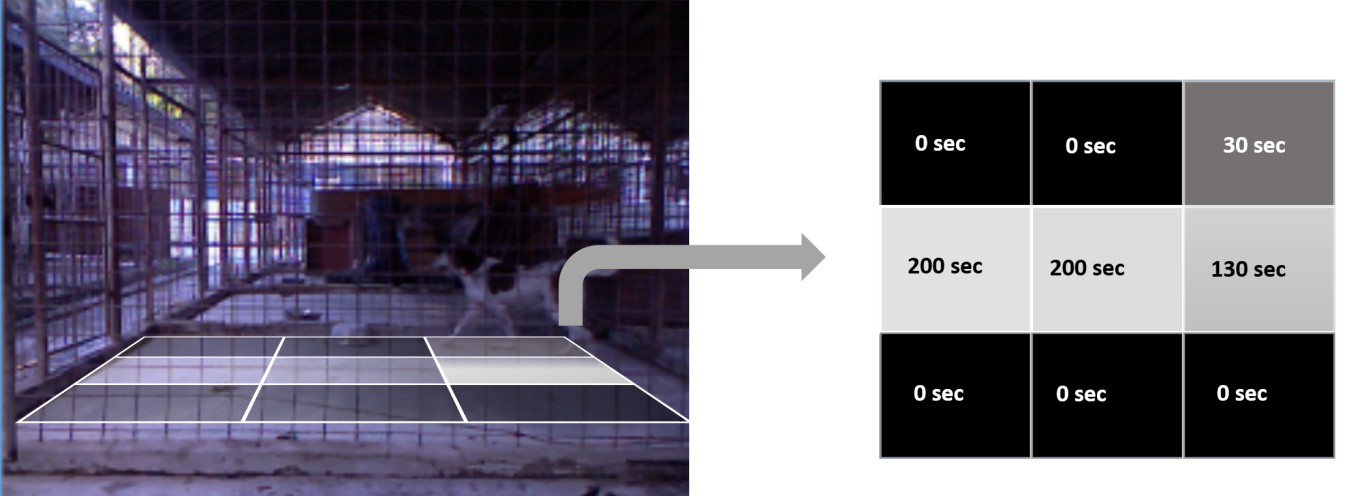


**Figure 7**: A grid divides the floor of the pen. The time (seconds) spent by the dog on each square is calculated. Different scales of grey also quantify the amount of time spent in each square (i.e. black = never entered the square, lighter shades = more time spent in that square).
