# Supplementary material for: Quick, Accurate, Smart: 3D Computer Vision Technology Helps Assessing Confined Animals’ Behaviour
Source: PLoS One. 2016 Jul 14;11(7):e0158748. doi: 10.1371/journal.pone.0158748 (PMC4944961; doi:10.1371/journal.pone.0158748)
Supplement: S1 Supporting Article — Article describing the algorithms behind the early developmental phases of the software. Corresponds to reference [35]. (PDF) [file pone.0158748.s006.pdf]

2014 22nd International Conference on Pattern Recognition

Kernelized Structural Classification for 3D dogs body parts detection

Simone Pistocchi*, Simone Calderara*, Shanis Barnard†, Nicola Ferri† and Rita Cucchiara*
*Engineering Department "Enzo Ferrari", University of Modena and Reggio Emilia
Modena, Italy
Email: {name.surname}@unimore.it
†Istituto Zooprofilattico Sperimentale dell'Abruzzo e del Molise "G. Caporale",
Teramo, Italy
Email: {s.barnard,n.ferri}@izs.it

***Abstract*—Despite pattern recognition methods for human behavioral analysis has flourished in the last decade, animal behavioral analysis has been almost neglected. Those few approaches are mostly focused on preserving livestock economic value while attention on the welfare of companion animals, like dogs, is now emerging as a social need. In this work, following the analogy with human behavior recognition, we propose a system for recognizing body parts of dogs kept in pens. We decide to adopt both 2D and 3D features in order to obtain a rich description of the dog model. Images are acquired using the Microsoft Kinect to capture the depth map images of the dog. Upon depth maps a Structural Support Vector Machine (SSVM) is employed to identify the body parts using both 3D features and 2D images. The proposal relies on a kernelized discriminative structural classificator specifically tailored for dogs independently from the size and breed. The classification is performed in an online fashion using the LaRank optimization technique to obtaining real time performances. Promising results have emerged during the experimental evaluation carried out at a dog shelter, managed by IZSAM, in Teramo, Italy.**

***Keywords*—Structural learning, Dog Behavior, Body Part Recognition**

I. INTRODUCTION

Stray dog populations represent a serious concern for human beings health and safety and for dogs themselves in many European countries [1]. The main population control action plan in Italy, but also in other countries, is the confinement of stray dogs in shelter facilities until re-homing. Unfortunately, the impairment between entrance and adoption rates often leads to an overcrowded scenario where dogs are likely to spend most part of their lives. Previous literature has shown how long-term confinement in shelters has detrimental effects on dogs' welfare [2], [3]. Behavioral responses are a direct reflection of an animal's attempts to cope with its environment. Failure in these coping strategies may lead to a reduction in the expression of normal behaviors, and an increase of abnormal or repetitive behaviors. The study of behavior as an indicator of poor welfare is therefore critical when assessing the well-being of shelter dogs. Video-recording and subsequent image analysis is far the most applied technique, because of its non-invasiveness. However, manual or semi-automatic methods of image scoring are very time consuming, and may show drawbacks since they rely on the observer subjectivity, sensitivity and level of accuracy. An automatic image recording system would allow to collect a bigger amount of data, operating also over long periods of time, with high precision and nonetheless saving precious human labor. Therefore, the aim of the paper is to propose an innovative and, to our knowledge, unique framework for measuring, in an automatic way, the behavioral parameters of dogs kept in kennel environment.

Computer vision analysis of dog body-parts is absent in literature, only a few experiments exist on animals, mostly involving livestock and focusing in classifying animals motion patterns. Shao et al. [4] analyzed the thermal comfort behavior of swine using programmable cameras and information based on the top view of the animals. Tillett et al. [5] applied image processing techniques to pigs in a pen in order to track their movement and extract information about position, rotation, bending and head nodding with the aim of studying their individual behavior. Leroy et al. [6] developed a model-based computer vision system to study the behavior of hens to assess their welfare degree. Analyzing the hens contour they extract the posture and classify the possible behaviors into predetermined categories (i.e. as "standing", "walking", "scratching", ...). Cangar et al. [7] developed an automatic image analysis system able to identify some locomotion and posture behaviors of cows prior to calving with the purpose of alarming when a human intervention is necessary. However, all of these works are often oversimplified and operate in supervised and controlled settings. The complexity of real scenarios poses new challenges and emerges the need to precisely analyzing animals complex behaviors in order to evaluate their welfare in real situations. To this aim we believe it is of broad interest to provide details about quadrupeds posture or body parts. To our knowledge there are no proposal about this topic. Conversely, most of the existing works about body parts detection focus on human beings. Most of the solutions relies on the analysis of 2D images where features are analyzed using pre-trained classifier either generative [8], [9] or discriminative [10]. Conversely 3D approaches have exploited the richness of the three dimensional representation that conveys important information able to solve partial occlusions between body parts, [11]. Among the 3D sensors the Microsoft Kinect sensor have been profitably exploited for body part detection and tracking, [12]. Recently, Structural classifiers have emerged as a valuable tool for body parts detection. Structural classification exploit at the same time

1051-4651/14 $31.00 © 2014 IEEE
DOI 10.1109/ICPR.2014.348

IEEE computer society

body parts model and their mutual relation in a joint classification framework, [13]. The main flaws of structural classification reside in the need of an exhaustive training set and the computational cost of the classification algorithm that prevents a real time application.

Although techniques that explicitly model the human body cannot be directly applied to quadrupeds, we propose the adoption of a structural classifier to detect dogs body part. Our proposal relies on the adoption of an efficient on-line training technique that allows the classification to be both real-time and widely applicable by the use of kernel functions. Our solution is the first tentative of identifying dogs body parts considering that dogs bodies have a completely different structure w.r.t. human ones (i.e. different axes of symmetry, a different kind of self occlusions, different motion constraints ...). The adoption of 3D features allows the method to be invariant to the dog breed and size while it can be easily extended to different quadrupeds body models by changing the kernel functions leaving the rest of the proposal unchanged.

II. Structural classification of dog body parts

The method for body part classification is constituted by two different steps; first the dog is located inside the scene and its image is extracted, second depth features are extracted and eventually classified. We restricted the dog body model as being constituted by seven body parts (torso, head, tail and the four paws) and considered pens containing one animal alone. This hypothesis simplifies the detection and tracking problem. Nevertheless, the dog tracking can be conducted, even in complex scenarios, with a single target tracker. For further details readers can refer to the survey in [14]. Considering the structure of the pen, depth maps, acquired by the Kinect sensor (Fig. 1.b) are exploited to remove the planes that delimit the pen itself with a least-square plane fitting method. After planes removal, only the blob containing the dog remains, Fig. 1.c. Finally, morphological binary operators are applied to the dog mask to fill potential holes due either to noise or Kinect errors (Fig 1.d) and both the dog depth image and the distance transform, computed on the dog binary mask, are extracted as the features for classification, Fig. 1.(e-f).

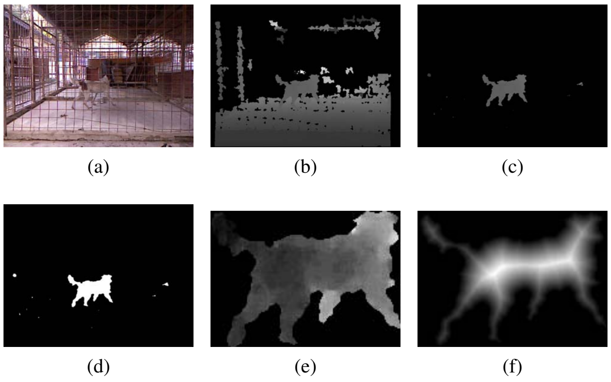

Fig. 1. (a) Color image. (b) Depth image. (c) Dog body after pen planes removal. (d) Dog Blob. (e) Dog Depth image. (f) Dog Distance Transform image.

A. Structural Support Vector Machine

We formulate the problem of dog body-part detection as a structured learning problem using Structural Support Vector Machine (SSVM) [15].
SSVM represents an effective solution for structural learning problem and has been profitably applied in different computer vision context from segmentation [16] to tracking [17]. The use of a structural classifier allows to consider jointly the body parts model and their inner relationships derived from anatomical constraints leading to an accurate classification without the need of explicitly define the body model itself.
The classification is performed frame by frame. Let us consider an input vector $x \in \mathcal{X}$ that represents the dog features and a set of possible solutions (i.e. body parts labeling) $\mathcal{Y}$. In a supervised discriminative setting, the classifier aims to learn a classification function $h : \mathcal{X} \to \mathcal{Y}$ based on training samples of input-output pairs. This function is expressed as the maximization of a discriminant function $F : \mathcal{X} \times \mathcal{Y} \to \mathbb{R}$:

$$h(x) = \underset{y \in \mathcal{Y}}{argmax}\; F(x, y) \tag{1}$$

that measures the compatibility between (x, y) pairs, returning a high score value for well-matched pairs.
The training of the classifier is performed by parametrizing the scoring function of Eq. (1) by a weight vector w and expressing F as a dot product between w and a joint kernel map $\Psi(x, y)$ that maps an input output pair (x, y) to a real valued features vector, $F(x, y) = \langle w, \Psi(x, y)\rangle$. This Structured Support Vector Regression problem is solved by estimating the parameter vector w in a loss augmented learning setting where the loss function $\Delta(y, \bar{y})$ measures the difference between two possible solutions y and $\bar{y}$. The objective here is learning w so that the value of $F(x_i, y_i) - F(x_i, y)$ mimics as close as possible the loss function behavior $\Delta(y_i, y)$. Basing on a set of sample pairs $\{(x_1, y_1), \dots, (x_n, y_n)\}$, during training we solve the dual SSVM convex optimization problem in its re-parametrized form proposed in [18]:

$$\begin{aligned} &\max_{\beta} - \sum_{i,y} \Delta(y, y_i)\beta_i^y \;-\; \frac{1}{2}\sum_{i,y,j,\bar{y}} \beta_i^y \beta_j^{\bar{y}} \langle \Psi_i(x_i, y), \Psi_j(x_j, \bar{y})\rangle \\ &s.t. \quad \forall i, \forall y : \beta_i^y \leq \delta(y, y_i)C \\ &\qquad\; \forall i : \sum_{y \neq y_i} \beta_i^y = 0 \end{aligned} \tag{2}$$

where

$$\delta(y, y_i) = \begin{cases} y_i & \text{if } y = y_i \\ 0 & \text{otherwise} \end{cases} \tag{3}$$

The discriminant function then becomes:

$$F(x, y) = \sum_{i,\bar{y}} \beta_i^{\bar{y}} \langle \Psi(x_i, \bar{y}), \Psi(x, y)\rangle. \tag{4}$$

In Eq. (4) the joint kernel map Ψ does not need to be defined explicitly because it appears only inside a dot product operation thus the problem can be solved using a kernel function $K(x_i, y_i, x_j, y_j)$ instead, as in the dual SVM case. In a similar vein, the pairs (x_i, y) having $\beta_i^y \neq 0$ are considered as the *support vectors*. The support vectors having $\beta_i^y > 0$ are referred as *positive* because they contribute positively to

Algorithm 1 SMO Step

Require: $i,\ y_+,\ y_-$

$k_{00} = \langle \Psi(x_i, y_+), \Psi(x_i, y_+)\rangle$

$k_{11} = \langle \Psi(x_i, y_-), \Psi(x_i, y_-)\rangle$

$k_{01} = \langle \Psi(x_i, y_+), \Psi(x_i, y_-)\rangle$

$\lambda^u = \frac{g_i(y_+) - g_i(y_-)}{k_{00} + k_{11} - 2k_{01}}$

$\lambda = max(0, min(\lambda^u, C\delta(y_+, y_i) - \beta_i^y))$

Update coefficients

$\beta_i^{y+} \leftarrow \beta_i^{y+} + \lambda$

$\beta_i^{y-} \leftarrow \beta_i^{y-} - \lambda$

Update gradients

for $(x_j, y) \in S$ **do**

  $k_0 = \langle \Psi(x_j, y), \Psi(x_i, y_+)\rangle$

  $k_1 = \langle \Psi(x_j, y), \Psi(x_i, y_-)\rangle$

  $g_j(y) \leftarrow g_j(y) - \lambda(k_0 - k_1)$

end for

the discriminant function, conversely, those having $\beta_i^y < 0$ are referred as *negative*. Those x_i that are included in at least one support vector are defined as *support patterns*.

The use of kernels in structural SVM is an advantage because it allows, similarly to SVMs, the classification function to be non linear, but the computational cost of the dual problem increases as it involves evaluating the kernel for every support vector [19].
To overcome this problem we make use of the iterative Sequential Minimal Optimization(SMO) technique proposed in [18], LaRank.

B. LaRank

LaRank algorithm is a stochastic learning algorithm used to estimate the coefficient β of the constrained optimization problem of Eq. (2), that combines partial gradient information with the randomization arising from the sequence of training examples. Differently from optimization algorithms that rely on the evaluation of the full solution space, LaRank performs a randomized exploration inspired by subgradient methods. The algorithm is based on a sequence of SMO-style steps, [20]. At every step, it modifies a pair of coefficients β_i^{y+} and β_i^{y-}, by adding and subtracting a fixed quantity λ to fulfill the constraint $\sum_y \beta_i^y = 0$. This constitutes a one-dimensional maximization problem in λ that can be solved using the SMO technique, see Alg. 1. Gradients g_i in Alg.1 are computed for a single coefficient β_i^y, as:

$$\begin{aligned} g_i(y) &= -\Delta(y, y_i) - \sum_{j,\bar{y}} \beta_j^{\bar{y}} \langle \Psi_i(x_i, y), \Psi_j(x_j, \bar{y})\rangle \\ &= -\Delta(y, y_i) - F(x_i, y). \end{aligned} \tag{5}$$

LaRank considers three different update strategies for choosing y_+ and y_- in Alg.1.

The *PROCESSNEW* step processes a new training sample (x_i, y_i) and selects $y_+ = y_i$ and $y_- = argmin_{y\in Y} g_i(y)$. This step adds the correct solution (x_i, y_+) as a positive support vector and search for the worst solution (x_i, y_-) as the corresponding negative support vector. It is important to notice that a new support vector is not created if the SMO-step doesn't modify the β coefficients.

Instead, the *PROCESSOLD* step processes an existing support pattern x_i, chosen randomly, where $y_+ = argmax_{y\in Y} g_i(y)$ with $\beta_i^y < \delta(y, y_i)C$ and $y_- = argmin_{y\in Y} g_i(y)$. This step revisits an existing positive support vector possibly adding (x_i, y_-) as a new negative example.
Lastly the *OPTIMIZE* step processes an existing support pattern x_i, chosen randomly among the existing support vectors, setting $y_+ = argmax_{y\in Y} g_i(y)$ with $\beta_i^y < \delta(y, y_i)C$ and $y_- = argmin_{y\in Y_i} g_i(y)$ with $Y_i = \{y \in Y | \beta_i^y \neq 0\}$.
The algorithm doesn't specify a termination criterion. As suggested in [18], we schedule the update steps as follows: given a new training sample (x_i, y_i) we invoke a PROCESSNEW followed by η_R REPROCESS, defined as a PROCESSOLD followed by η_O OPTIMIZE. We set η_O = η_R = 10.

C. Kernel and Loss functions for dogs body parts

We define a solution y of (1) as a 14 dimensional vector containing the image coordinates of the segments that represent the body part in the following order: torso, head, tail front left paw, front right paw, bottom left paw and bottom right paw as can be seen in Fig. 2.

a) Kernel Function: Every input-output pair (x_i, y_j) generates a feature vector based on the mapping $\Psi : \mathcal{X} \times \mathcal{Y} \rightarrow \mathbb{R}^d$. For our problem, we use features derived both from the depth map, obtained from the Kinect sensor, and the distance transform computed on the binary mask of the dog body. The distance transform maps binary images into gray-scale images replacing every pixel of the object with its distance from the nearest pixel of background, Fig. 1(e-f).

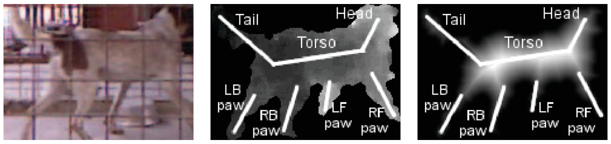

(a) (b) (c)

Fig. 2. (a) Dog RGB image. (b-c) Solution y drawn over the depth image and the Distance Transform image respectively.

In particular we choose the mean and the variance of the depth and the distance transform values along the segments that identify the body parts in the solution vector y, Fig. 2(b-c). These appear to be simple and effective descriptors because the distance transform allows us to obtain a sketch of the skeleton of the dog while the depth image, instead, helps to distinguish among paws as resulted from our experiments. We additionally add the components of the motion vector of the dog barycenter computed between two consecutive frames in order to support the system to point the torso in the correct direction; finally we obtain a 16 dimension real valued vector $\varphi(x, y)$. Given two input-output pairs the kernel function $K(\Psi(x_i, y_i), \Psi(x_j, y_j))$ is then computed using RBF Gaussian Kernel with $\sigma^2 = 1$:

$$K(\Psi(x_i, y_i), \Psi(x_j, y_j)) = exp(-\|\varphi(x_i, y_i) - \varphi(x_j, y_j)\|^2) \tag{6}$$

b) Loss Function: The Loss function $\Delta : \mathcal{Y} \times \mathcal{Y} \rightarrow \mathbb{R}$ in eq. (2), as previously stated, is used during training to evaluate the dissimilarity between two solutions.
We derive the loss function as the inverse of the PCP measure

(Percentage of Correctly estimated body Parts) of Eichner and Ferrari [21]. The PCP measure is a well assessed measure to evaluate the accuracy in human body parts classification systems. The PCP value is based on the criterion that a body part is considered correctly estimated if its segment endpoints lie within of the length of the ground-truth segment from their annotated locations. The loss function, $\Delta(y, y')$, is then computed as the inverse of the PCP between two different solutions:

$$\Delta(y, y') = 2(1 - PCP(y, y')) \tag{7}$$

III. Solution generation for quadrupeds body structure

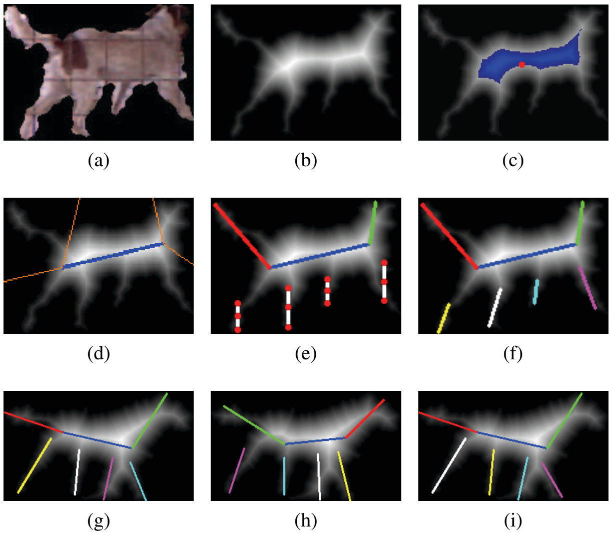

Fig. 3. (a) Color image. (b) DT on dog mask (c) Torso detection area. (d) Angular constraint for head and tail (e) Initial paws. (f) Refined paws. (g-i) Solutions obtained iterating the procedure and after labels permutation.

Once the SSVM is correctly trained, inference on the solution vector y is obtained by maximizing the discriminant function of Eq. (1) using Eq. (4). The maximization process involves the generation of possible feasible solution vectors $y \in \mathcal{Y}$ in order to compute the *argmax* operation. In the case of body part detection, this involves considering all possible dog poses w.r.t the camera that is not feasible due to the complexity of the articulated motion of quadrupeds. This limitation has been overcome designing a heuristic method for the solution generation process where possible solutions y are generated considering the distance transform DT computed on the dog binary mask, as in Sec. II-C. In detail, the i-th body part segment s_i is described by the quadruple $\{x_{0i}, y_{0i}, \theta_i, L_i\}$ where (x_0, y_0) is one of the extreme of the segment, θ the angle between an horizontal line and the segment (computed counterclockwise) and L the length of the segment. Let n be the number of the searched body parts and $S = \{x_{0k}, y_{0k}, \theta_k, L_k\}_{k=1}^{n}$ be the body parts segments; we formulate the problem of finding possible solutions as finding the sets of segments that maximizes the sum of DT along their points:

$$S_j = \underset{S}{argmax} \sum_{k=1}^{n} \sum_{(x,y)\in s_k} DT(x, y) \tag{8}$$

We constrained the set of possible solution segments S using quadrupeds anatomical constraints. The first set of constraints involves the top portion of the body parts, namely the torso, head and tail. We force the torso segment to pass through the dog barycenter, lying inside the area where the distance transform reaches its maximum values, Fig. 3.c. Head and tail then start respectively from the start point and the endpoint of the torso and we additionally impose an angular constraint on the search space of segment parameters of 150 degrees, w.r.t the torso direction, as shown in Fig. 3.d.

Finally the paws are constrained in the area beyond the torso segment. We heuristically search for the set of possible solutions by iteratively finding, for every body part segment, its global maximum of Eq. (8) until a complete solution, that involves all the seven body parts, is built.

The complete heuristics procedure is visually sketched in Fig. 3. First the torso is searched using the aforementioned constraint. Then head and tail segments are scanned starting from torso endpoints inside the limited angular area. Lastly the paws are extracted. We first scan the area below the torso using four fixed-size vertical segments until a local maximum of Eq. (8) is found, Fig. 3.e. The paws are then refined shrinking and rotating the segments w.r.t. segments midpoints, starting points and endpoints until the maximum value of DT along segments is reached, Fig. 3.f. To generate a set of possible solutions, we iterate N times the procedure removing the previous segments from the DT image. After the set of N possible solution segments is computed we finally create the y vectors assigning to every segment its label. A label permutation step between head and tail, and accordingly to paws, is employed to account, in the solution generation process, for different dog orientations. This method covers a very large portion of possible orientation of the torso showing some shorcomings only when the dog is exactly front or back to the camera. An example of possible solution computed by this heuristic procedure is depicted in Fig. 3 (g-i).

IV. Experimental Results

In order to perform tests, we acquired a specific dataset of dog videos in a kennel environment. We remark that there is no presence of publicly available datasets of this kind. Two different real scenarios have been considered depending whether the dog pen is indoor or outdoor that affects the lighting condition during the shooting of the videos. In particular two test trials have been performed in the italian kennel, Test. 1 and Test. 2. An additional test trial Test 3 was performed in a fully controlled environment in our laboratory.

Trial. 1 contains sequences acquired with constant lighting conditions. Conversely Trial. 2 exhibits severe difference in the lighting of the pen. All the test involved different breeds of dogs.

Videos have been shooting using the Microsoft Kinect at a distance of one meter from the pen. The pens size have been restricted to a maximum of 3 meter width and 3 meters from the sensor. This restriction is due to the Kinect operation range but the method can be applied to wider pens using a stereo camera instead. All the trials involves varying length sequences for a total recording time of half an our per trial at 10 frame per second.

The total number of frames considered for classification are 8340 for Trial 1, 8110 for Trial 2 and 6000 for Trial 3; in all the

TABLE I. RESULTS OF OUR EXPERIMENTS FOR VARIOUS TRAINING SET.

Training Size	67	128	228	385
Trial 1	55.9%	64.1%	67.7%	**70.3%**
Trial 2	49.4%	52.7%	58.4%	**67.4%**
Trial 3	52.2%	68.3%	77.5%	**78.2%**

TABLE II. PCP VALUES FOR TOP & BOTTOM BODY PARTS. CLASSIFICATION

	top body parts	bottom body parts
Trial 1	82.6.8%	65.6%
Trial 2	78.4%	63.7%
Trial 3	87.3%	74.5%

TABLE III. COMPARISON OF OUR SOLUTION WITH THE SSVM HUMAN BODY POSE CLASSIFICATION ALGORITHM IN [13]

	Our Proposal	SSVM in [13]
Trial 1	**70.3%**	50.4%
Trial 2	**67.4%**	48.3%
Trial 3	**78.2%**	53.4%

TABLE IV. QUALITATIVE EVALUATION ON THE COMPLETE DATASET.

	Correct Solution	Partially Correct Solution	Mostly Wrong Solution
Trial 1	74.5%	20.4%	5.1%
Trial 2	70,5%	28.5%	1.0%
Trial 3	88.1%	9.8%	2.1%

frames the dogs are present and awaken. After the acquisition, a 10% of frames, randomly chosen for every test trial, have been manually annotated and used for quantitative evaluation, while the remaining frames have been evaluated qualitatively by three experts and detected parts have been labeled as either correct or wrong based on majority voting.
The effectiveness of the heuristic for solution generation, described in Sec. III has been evaluated on the manually annotated portion of the dataset. It was considered the number of frames in which the algorithm was able to generate at least one correct solution and this happened in the 97,7% of the frames we tested while the number of correct solution with a maximum of two mislabeled body parts reached the 100%.

The classification accuracy was evaluated using the PCP measure [21], the same measure we exploited for computing the loss function of the SSVM in Sec. II-C0b.
The PCP is state-of-the-art performance measure for the human body pose estimation problem and can be directly employed for quadrupeds without any further modification.
We trained the classifier choosing randomly the input-output pairs (x, y) among the manually annotated images. In principle, different test have been performed varying the training set size while the C parameter of the SSVM have been set by grid-search. Quantitative results, in term of PCP, for the three test trials on the annotated frames are shown in Tab. I.

It was noted that the lower performance in Trial 2 are mostly due to the sensitivity of the Kinect to strong illumination changes that resulted in dog masks with many holes and imprecise depth images. We perform an additional test to underline which body part are mostly mislabeled calculating the PCP for the top body parts (torso, head and tail) and for the bottom ones (the 4 paws), Tab. II. Observing the results we noted that paws are more frequently wrongly classified w.r.t. the other body parts. Most of the errors involves the swapping of the paws closer to the camera with the farther ones mainly when depth images are imprecise or noisy due to sensor inaccuracies. That problem can be partially mitigated by the adoption of a stereo camera with a higher resolution than the Kinect, increasing the costs of the system.
Since no methods exist for dog body part classification we compare our system against the proposal in [13], that employs SSVM for human body pose estimation. In order to perform the comparison, kernels have been set equal for both the tested methods while the solution generation process and the loss function have been varied according to [13].

The comparison was performed on the annotated part of the dataset and the results shown in Tab. III demonstrate that both the heuristic described in Sec. III and the loss function are specifically tailored for quadrupeds classification leading to more accurate results.

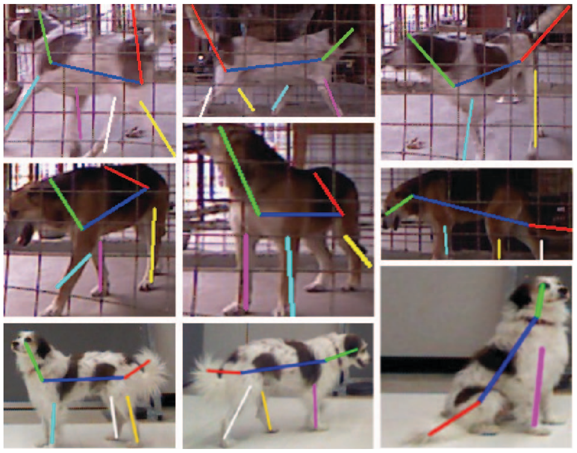

Fig. 4. Visual results on the trial scenarios. First row are images from Trial 1 sequences, Second and third rows are from Trial 2 and 3 respectively.

Finally qualitative tests have been performed on the complete dataset. We classified all the frames automatically and for every solution we asked three experts to evaluate the classification results using three classes (in the case of discordance among experts we use majority voting):

- Correct Solution: where body parts appear visually correct.
- Partially Correct Solution: where at least a half of the body parts appears visually correct.
- Mostly Wrong Solution: where visually the body parts are perceived as wrongly detected.

The qualitative performances in Tab. IV are higher than the quantitative ones because PCP measure accounts for the

precise localization of the body part. Nevertheless, experts agree that only an average 4% of the images are completely misclassified. Visual results obtained by our proposal on three different dog breeds in the trial scenarios can be observed in Fig. 4.

V. Conclusions

We presented a system that approaches the novel problem of dog body parts detection using a 3D sensor. The 3D depth images are acquired by the Microsoft Kinect sensor and used, in conjunction with the distance transform values, to effectively classify the dog body parts. The adoption of a structural classifier allows to capture the relation among body parts without explicitly modeling all the anatomical constraint in the dog body. During experiments, carried out on dogs kept in kennel, we observe promising results of the system both in terms of the quantitative PCP measure and the qualitative visual evaluation. Tests have exhibited the independence of the proposal w.r.t to dog breeds, and we expect it being applicable to different kind of quadrupeds without excessive changes. We believe that this can constituts a first important step for analyzing dog behavior in kennels in order to detect repetitive and other aberrant behaviors, common indicators of poor welfare for confined animals.

Acknowledgment

The study was founded by the Italian Ministry of Health through the research project IZS AM 01/11 RC "Sistemi Tecnologici per il controllo delle popolazioni Canine".

References

[1] P. DallaVilla *et al.*, "Free-roaming dog control among oie-member countries," *Preventive Veterinary Medicine*, vol. 97, no. 1, pp. 58 – 63, 2010.

[2] D. Wells, L. Graham, and P. Hepper, "The influence of length of time in a rescue shelter on the behaviour of kenneled dogs," *Animal Welfare*, no. 11, 2002.

[3] K. Taylor and D. Mills, "The effect of the kennel environment on canine welfare: a critical review of experimental studies." *Animal Welfare*, no. 16, 2007.

[4] J. Shao, H. Xin, and J. Harmon, "Comparison of image feature extraction for classification of swine thermal comfort behavior," *Comput. Electron. Agric.*, vol. 19, no. 3, p. 223232, 1998.

[5] R. Tillett, C. Onyango, and J. Marchant, "Using model-based image processing to track animal movements: livestock monitoring," *Comput. Electron. Agric.*, vol. 17, no. 2, pp. 249–261, 1997.

[6] T. Leroy *et al.*, "A quantitative computer vision method for on-line classification of poultry behavior in furnished cages," *Trans. ASAE*, vol. 49, no. 3, pp. 795–802, 2005.

[7] O. Cangar *et al.*, "Automatic real-time monitoring of locomotion and posture behaviour of pregnant cows prior to calving using online image analysis," *Computers and Electronics in Agriculture*, vol. 64, pp. 53–60, 2008.

[8] P. Felzenszwalb and D. Huttenlocher, "Pictorial structures for object recognition," *International Journal of Computer Vision*, vol. 61, no. 1, 2005.

[9] L. Bourdev and J. Malik, "Poselets: Body part detectors trained using 3d human pose annotations," in *In Proc. ICCV*, 2009.

[10] G. Gkioxari, P. Arbelaez, L. Bourdev, and J. Malik, "Articulated pose estimation using discriminative armlet classifiers," in *Proceedings of the 2013 IEEE Conference on Computer Vision and Pattern Recognition*, ser. CVPR '13, 2013.

[11] E. Simo-Serra, A. Ramisa, G. Alenya, C. Torras, and F. Moreno-Noguer, "Single image 3d human pose estimation from noisy observations," in *Computer Vision and Pattern Recognition (CVPR), 2012 IEEE Conference on*, 2012, pp. 2673–2680.

[12] J. Shotton *et al.*, "Real-time human pose recognition in parts from single depth images," in *Proceedings of the 2011 IEEE Conference on Computer Vision and Pattern Recognition*, ser. CVPR '11. Washington, DC, USA: IEEE Computer Society, 2011, pp. 1297–1304. [Online]. Available: http://dx.doi.org/10.1109/CVPR.2011.5995316

[13] K. Chen, S. Gong, and T. Xiang, "Human pose estimation using structural support vector machines," in *Proceedings of the 2011 IEEE International Conference on Computer Vision*, 2011.

[14] A. Smeulder *et al.*, "Visual tracking: an experimental survey," *IEEE Transactions on Pattern Analysis and Machine Intelligence*, Nov. 2013.

[15] T. Joachims, T. Finley, and C.-N. Yu, "Cutting-plane training of structural svms," *Machine Learning*, vol. 77, no. 1, pp. 27–59, 2009.

[16] L. Bertelli, T. Yu, D. Vu, and B. Gokturk, "Kernelized structural svm learning for supervised object segmentation," in *Computer Vision and Pattern Recognition (CVPR), 2011 IEEE Conference on*, 2011, pp. 2153–2160.

[17] S. Hare, A. Saffar, and P. H. Torr, "Struck: Structured output tracking with kernels," in *Proceedings of the 2011 IEEE International Conference on Computer Vision*, 2011.

[18] A. Bordes, L. Bottou, P. Gallinari, and J. Weston, "Solving multiclass support vector machines with larank," in *Proceedings of the 24th International Machine Learning Conference*, Z. Ghahramani, Ed. Corvallis, Oregon: OmniPress, 2007, pp. 89–96. [Online]. Available: http://leon.bottou.org/papers/bordes-2007

[19] C.-N. Yu and T. Joachims, "Training structural svms with kernels using sampled cuts," in *ACM SIGKDD Conference on Knowledge Discovery and Data Mining (KDD)*, 2008, pp. 794–802.

[20] J. C. Platt, "Fast training of support vector machines using sequential minimal optimization," *MIT Press*, p. 185208, 1999.

[21] M. Eichner and V. Ferrari, "Better appearance models for pictorial structures," in *Proceedings of British Machine Vision Conference*, 2009, p. 111.
